# Alteplase in COVID-19 severe hypoxemic respiratory failure: the TRISTARDS multicenter randomized trial

**DOI:** 10.1186/s13613-024-01386-z

**Published:** 2024-11-10

**Authors:** Giovanni Landoni, Pratima Chowdary, Ferhat Meziani, Jacques Creteur, Nicolas De Schryver, Johann Motsch, Ingrid Henrichmoeller, Alain Pagès, Nuala Peter, Thierry Danays, Markus A. Weigand, the TRISTARDS Investigators, Alexander Zoufaly, Tamara Seitz, Erich Pawelka, Stephanie Neuhold, Wolfgang Höpler, Benedikt Rumpf, David Totschnig, Rudolf Likar, Markus Koestenverger, Stefan Neuwersch-Sommeregger, Jacques Creteur, Amedee Ego, Anthony Moreau, David Grimaldi, Filippo Annoni, Julie Gorham, Katarina Halenarova, Zoe Pletschette, Alexandre Brasseur, Fabio Taccone, Leda Nobile, Olivier Lheureux, Morgane Snacken, Charles Dehout, Nicolas De Schryver, Marco Vinetti, Nicolas Serck, Thierry Dugernier, Nathalie Layios, Gilles Parzibut, Rodrigo Boldo, Vanessa Santos dos Santos, Cristine Erdmann Nunes, Jean-Luc Diehl, N Aissaoui, J Augy, E Guerot, C Hauw-Berlemont, B Hermann, N Peron, F Santi, J Langlais, A Troger, K Chekhrit, Julien Poissy, M Caplan, A El Kalioubie, R Favory, A Gaudet, J Goutay, S Preau, A Rouze, Laure Mariller, Jean-Paul Mira, Z Ait Hamou, S Ben Ghanem, M Bertrix, J Charpentier, T Creutin, M Jozwiak, D Laghlam, E Peju, F Pene, C Vigneron, Ferhat Meziani, J Demisselle, J Helms, L Jandeaux, C Kummerlen, H Merdji, A Monnier, H Rahmani, A Studer, S Cunat, Ouafa Hakkari, Xavier Monnet, I Adda, N Anguel, S Ayed, Q Fosse, L Guerin, D Osman, A Pavot, T Pham, C Carpentier, P Denormandie, C Lai, Alain Fourreau, Mehran Monchi, O Ellrodt, S Jochmans, S Mazerand, N Rolin, J Serbource-Goguel, P Soulier, O Sy, Nourdine Benane, Bruno Mourvillier, J Cousson, A Goury, O Passouant, G Thery, Cédric Castex, Jean-Baptiste Lascarrou, A Roquilly, E Canet, C Garret, J Lemarie, M Martin, J Reignier, A Seguin, O Zambon, P Lamouche Wilquin, M Agbakou, P Decamps, L Desmedt, G Blonz, Y Hourmant, N Grillot, A Rouhani, M Bouras, P.-J. Mahe, D. Demeure Dit Latte, A Bourdiol, N Benkalfate, M Carpentier, F Guillotin, S Benguerfi, Johann Motsch, Johannes Zimmermann, Karam Al Halabi, Marc Altvater, Sebastian Decker, Mascha Fiedler, Phillip Knebel, Barbara Maichle, Markus Weigand, Tobias Welte, Nora Drick, Isabelle Pink, Julius Johannes Schmidt, Sven Bercker, Philipp Simon, Falk Fichtner, Gunther Hempel, Peter Kliem, Karsten Kluba, Sven Laudi, Sarah Müller, Rene Oesemann, Michael Roedel, Stefan Schering, Sebastian Schulz, Christian Seeber, Hannah Ullmann, Svitlana Ziganshyna, Nora Jahn, Bastian Boerge, Maren Keller, Michael Irlbeck, Sandra Frank, Ursula Hoffmann, Aydin Huseynov, Simone Britsch, Gill Ishar-Singh, Claude Jabbour, Sven Stieglitz, Jan-Erik Guelker, Maurizio Cecconi, Massimiliano Greco, Giacomo Monti, Maria Luisa Azzolini, Beatrice Righetti, Francisco Marquez Diaz, Sofía Elizabeth Girón, Alejandra Aviles de La Cruz, Ana Elena Ramírez Ibarra, Paola Hernández Romo, Marián Serna García, Andrés García Castillo, Peter Spronk, Marnix Kuindersma, Michiel Blans, Henk Leeuwen, Marco Peters, Els Rengers, Oscar Hoiting, Viktor Borisovich Filimonov, Maria Peshenniokva, Olga Kravchenko, Yuri Karev, Anastasiia Filimonova, Sergey Nikolaevich Avdeev, Svetlana Chikina, Tatiana Gneusheva, Zamira Merzhoeva, Galina Nekludova, Denis Nikolaevich Protsenko, Igor Tyurin, Nikita Matyushkov, Tatiana Valerievna V. Lisun, Aleksandr Boyarkov, Svetlana Bobkova, Alexey Klinov, Dmitry Schukarev, Nikolay Smolin, Ricard Ferrer, Xavier Nuvials, Sofia Contreras, Alejandro Cortés, Mariel Rojas Lora, Rafael Sierra, Samer Alarbe, Ana Fernandez, Mario Contreras, María Dolores Freire, Jaume Revuelto, Mikel Celaya, Judith Marín, Francisco Parrilla, Purificación Perez, Rosana Muñoz, Emilio Diaz, Cristina Mora, Candelaria de Haro, Edgard Moglia

**Affiliations:** 1 Department of Anesthesia and Intensive Care, IRCCS San Raffaele Scientific Institute, Milan, Italy; 2 School of Medicine, Vita-Salute San Raffaele University, Milan, Italy; 3 Katharine Dormandy Haemophilia and Thrombosis Centre, Royal Free Hospital, London, UK; 4 Faculté de Médecine, Hôpitaux Universitaires de Strasbourg, Nouvel Hôpital Civil, Service de Médecine Intensive-Réanimation, Université de Strasbourg (UNISTRA), Strasbourg, France; 5 Department of Intensive Care, ULB Hôpital Erasme, Brussels, Belgium; 6 Intensive Care Unit, Clinique St-Pierre, Ottignies, Belgium; 7 Department of Anesthesiology, Heidelberg University Hospital, Heidelberg, Germany; 8 Therapeutic Area Cardiovascular Medicine, Boehringer Ingelheim International GmbH, Ingelheim, Germany; 9 Fifth Department of Medicine, Faculty of Medicine Mannheim, University of Heidelberg, Mannheim, Germany; 10 Boehringer Ingelheim, Biberach an Der Riss, Germany; 11 TDC, Aix en Provence, France

**Keywords:** Alteplase, ARDS, COVID-19, Severe hypoxemic respiratory failure, Thrombolysis

## Abstract

**Background:**

Pulmonary intravascular thrombus formation has been widely observed in patients with respiratory failure, for example, in patients with SARS-CoV-2 infection (COVID-19). The aim of this study was to evaluate the efficacy/safety of alteplase thrombolysis in COVID-19 severe hypoxemic respiratory failure. In this multicenter, open-label study, patients were randomized to receive alteplase (low- or high-dose) over 5 days plus standard of care (SOC), or SOC alone. The primary endpoint was time to clinical improvement (≥ 2-point decrease on WHO Clinical Progression Scale, or hospital discharge) up to Day 28. Secondary endpoints included all-cause mortality at Day 28, treatment failure at Day 28 and change in arterial oxygen partial pressure/fractional inspired oxygen (PaO_2_/FiO_2_) ratio at Day 6 versus baseline.

**Results:**

Sixty-nine patients were randomized to alteplase (low- or high-dose) and 35 to SOC; 65% were on high-flow oxygen or non-invasive ventilation at baseline. Median time to clinical improvement was 25 days in the alteplase group and > 28 days (median not reached) in the SOC group. All-cause mortality was 8/69 (12%) versus 10/35 (29%) in the alteplase versus SOC groups, respectively (unadjusted risk difference [RD], − 17% [95% confidence interval (CI) − 34 to 0], p = 0.047; adjusted RD, − 16% [95% CI − 31 to 1], p = 0.058). The PaO_2_/FiO_2_ ratio (mean [standard deviation]) increased by + 30 (84) mmHg in the alteplase group and decreased by − 12 (59) mmHg in the SOC group (adjusted mean difference vs. SOC, p = 0.052). Differences were greater in patients receiving high-dose alteplase, and in those not receiving invasive ventilation. Eighteen patients (26.1%) in the alteplase group discontinued treatment due to adverse events. Major bleeding was more frequent with alteplase than with SOC (9 vs. 0 patients); no bleeding was fatal. The study closed early due to insufficient patient recruitment.

**Conclusion:**

Alteplase was not associated with faster clinical recovery from COVID-19 severe hypoxemic respiratory failure. A numerical difference in survival and PaO_2_/FiO_2_ ratio was observed, particularly in patients not receiving invasive ventilation. These exploratory findings merit further investigation in larger patient cohorts that are adequately powered to confirm the hypotheses generated in this study regarding the impact of alteplase on treatment outcomes.

*Trial registration* ClinicalTrials.gov: NCT04640194 (November 23, 2020); https://clinicaltrials.gov/study/NCT04640194 (early discontinuation due to insufficient patient recruitment).

**Supplementary Information:**

The online version contains supplementary material available at 10.1186/s13613-024-01386-z.

## Background

Pulmonary intravascular thrombus formation associated with fatal outcomes has been widely observed in patients with respiratory failure, for example, in patients with severe acute respiratory syndrome (SARS-CoV) in 2003, Middle East respiratory syndrome (MERS-CoV) in 2012 and severe acute respiratory syndrome coronavirus 2 (SARS-CoV-2) in 2019 [1–3]. In 2020 and 2021, high numbers of patients with SARS-CoV-2 infection were admitted to hospital with hypoxemic respiratory failure. Many of these patients progressed to acute respiratory distress syndrome (ARDS), a life-threatening form of viral pneumonia with a high mortality rate [4–7].

Endothelial damage and coagulopathy are the pathophysiologic hallmarks of coronavirus disease 2019 (COVID-19) ARDS. Onset is marked by diffuse alveolar damage with epithelial and endothelial injury, causing impairment of gas exchange and accentuating the inflammatory process [8, 9]. In addition, increased tissue factor expression and suppression of fibrinolytic activity lead to a high risk of thrombosis, characterized by the formation of microthrombi (‘microclots’) in the lungs, brain and other vital organs [10–14]. In a systematic review of autopsies from 151 patients with COVID-19, 73% of cases had microthrombi in the lung, 11% in the heart, 24% in the kidney and 16% in the liver [15]. The presence of microthrombi in these patients was significantly associated with diffuse alveolar damage in exudative and proliferative phases [15], suggesting that anticoagulation and/or fibrinolytic drugs might be of benefit.

Small-scale exploratory studies and patient case series published during the pandemic supported the therapeutic potential of fibrinolytic drugs in improving microvascular patency, clinical outcomes and oxygenation in critically ill patients with COVID-19 [16–20]. As an established thrombolytic therapy [21–25], alteplase (recombinant tissue plasminogen activator) was hypothesized to improve outcomes in COVID-19- associated ARDS [26]. The TRISTARDS trial (ThRombolysIS Therapy for ARDS) aimed to evaluate the efficacy and safety of intravenous (i.v.) alteplase in patients with severe hypoxemic respiratory failure associated with COVID-19.

## Methods

### Trial design

TRISTARDS was a multinational, operationally seamless, open-label, randomized, sequential, parallel-group adaptive trial carried out at 34 sites in 10 countries (see Table S1 for list of sites and countries, and Figure S1 for study design). The study consisted of two parts: Part 1, an exploratory, dose-finding, proof-of-concept Phase IIb study; and Part 2, a confirmatory, Phase III study.

In Part 1, patients were randomized 1:1:1 to two dose regimens (low-dose or high-dose) of alteplase treatment added to standard of care (SOC), or SOC alone, for up to 5 days. The low-dose regimen included initial i.v. loading infusion of alteplase 0.3 mg/kg over 2 h (Day 1) followed by daily i.v. infusion of 0.02 mg/kg/h over 12 h. The high-dose regimen included initial i.v. infusion of alteplase 0.6 mg/kg over 2 h (Day 1) followed by daily i.v. long-term infusion of 0.04 mg/kg/h over 12 h. In Part 2, the high-dose regimen was carried forward based on evaluation of data from Part 1 by a Data Monitoring Committee (see supplementary material). Patients were randomized 2:1 to this dosing regimen of alteplase plus SOC, or SOC alone, with the aim of providing more critically ill patients with an active treatment that had a large potential benefit. Randomization in both study parts was stratified by ventilation status, and additionally D-dimer status in Part 2.

SOC represented the best possible treatment regimen established locally, in line with guidelines for the treatment of severe hypoxemic respiratory failure associated with COVID-19 at the time of the study (see supplementary material for treatments included in SOC). Thromboprophylaxis with anticoagulant therapies, either low doses of low-molecular-weight heparin (administered subcutaneously) or unfractionated heparin, was recommended for all patients in both groups to prevent the formation of new clots (see supplementary material for infusion scheme and details of preventative measures).

### Trial population

Both Parts 1 and 2 included patients with severe hypoxemic respiratory failure associated with SARS-CoV-2 infection (confirmed by reverse transcription polymerase chain reaction), who were mostly being treated in an intensive care unit (90% of enrolled patients). Severity of respiratory failure was classified according to the World Health Organization (WHO) Clinical Progression Scale [27] (all patients had a score of 6–9; see Table S2).

Inclusion criteria included: age ≥ 18 years (or above legal age); arterial oxygen partial pressure (PaO_2_)/fractional inspired oxygen (FiO_2_) ratio > 100 and ≤ 300; fibrinogen level ≥ lower limit of normal; D-dimer ≥ 3-fold upper limit of normal (ULN; Part 1) (modified to ≥ 1-fold ULN in Part 2) per local laboratory values; and written or verbal informed consent. For common oxygen delivery systems such as nasal cannulas and masks, FiO_2_ ranges were estimated from flow rates (Table S3). In addition, in situations where arterial blood gases were unavailable, PaO_2_/FiO_2_ ratio was inferred from oxygen saturation (Table S4). Exclusion criteria included: massive confirmed pulmonary embolism (PE) with hemodynamic instability, or suspected or confirmed PE that was expected to require therapeutic doses of anticoagulants; an indication for therapeutic dosing of anticoagulants; invasive mechanical ventilation (IMV) for longer than 48 h; and a history of chronic pulmonary disease, primary pulmonary arterial hypertension, bleeding disorder, or intracranial hemorrhage in the past 6 months. For full details, see supplementary material.

### Pooling of data and subgroup analyses

Due to study discontinuation, data from Parts 1 and 2 of the study were pooled for the main analysis. Thus, results are reported for all patients who received alteplase plus SOC versus all patients who received SOC alone (regardless of dose and/or ventilation status) for all primary and secondary endpoints common to both parts of the study, as well as for the further endpoint of all-cause mortality at Day 90. The pooled dataset therefore includes patients treated with both the low and high dose of alteplase in Part 1, and those treated with the high dose in Part 2.

Following the early discontinuation of the study, three analyses were conducted on the pooled data from Parts 1 and 2: the main analysis reported in this manuscript and Figure S2, and two subgroup analyses (supplementary analyses 1 and 2), which are presented in the supplementary material (for more detail, please see ‘Statistical analyses: Adjustments’ section and Table S5). These analyses were in line with the prespecified hierarchical testing approach planned for Part 2 but used pooled data from Parts 1 and 2 in the subgroup of patients not on invasive ventilation. The first subgroup analysis compares patients receiving high-dose alteplase with those receiving low-dose alteplase and SOC alone; the second includes all patients not receiving invasive ventilation, i.e. those with a WHO clinical score of 6, limited to high-dose alteplase, following the positive findings from Part 1 (see Tables S6 to S11 and Figures S3 to S7).

### Study endpoints

#### Primary endpoint

The primary endpoint in Parts 1 and 2 was time to clinical improvement up to Day 28, defined as the time from randomization to either an improvement of ≥ 2 points on the 11-point WHO Clinical Progression Scale (see Table S2) [27] or discharge from the hospital, whichever came first.

#### Secondary endpoints

In Part 1, secondary endpoints included: treatment failure (all-cause mortality or mechanical ventilation at Day 28); all-cause mortality (Day 28); number of ventilator-free days (Day 28); PaO_2_/FiO_2_ ratio change from baseline (daily average) (Day 6); and improvement of Sequential (sepsis-related) Organ Failure Assessment score by ≥ 2 points from baseline (Day 6).

In Part 2, the key secondary endpoints (per the hierarchical testing procedure) were treatment failure and all-cause mortality, both at Day 28. Other secondary endpoints included number of oxygen-free days (Day 28), length of hospital stay (Day 28) and PaO_2_/FiO_2_ ratio change from baseline (worst daily value) (Day 6). The occurrence of major bleeding events (MBEs, Day 6) was a secondary endpoint in both Parts 1 and 2. Major bleeds were defined according to the International Society on Thrombosis and Haemostasis definition [28] (for full definitions, see supplementary material).

Only secondary endpoints common to both Parts 1 and 2 are reported in this analysis. All-cause mortality at Day 90 was predefined as a further endpoint in both Parts 1 and 2.

### Safety monitoring

Bleeding events were monitored continuously. Unblinded safety data from Part 1 were evaluated by a Data Monitoring Committee to make a recommendation for proceeding with Part 2 and to select an appropriate dosing regimen.

### Statistical analyses

Primary, secondary and further endpoints were evaluated in an exploratory manner. All data were analyzed descriptively using standard methods, and most analyses were performed as both unadjusted and adjusted (treatment, baseline D-dimer status and ventilation status, age, and study part). Unadjusted analyses were prespecified in the statistical plan as sensitivity analyses, prior to the start of recruitment.

For the primary endpoint, patients were censored at Day 28 if they died or did not have clinical improvement prior to Day 28. Hazard ratios (HRs) were estimated from the Cox proportional hazards model and risk differences at Day 28 from the Kaplan–Meier (KM) estimates. Risk differences were estimated for the binary endpoints using logistic regression followed by the average marginal effect method, except for the safety endpoints, which used the Chan and Zhang method for the confidence interval (CI) determination, due to fewer events. For the Day 6 change in PaO_2_/FiO_2_ ratio, the mean difference was estimated between the two groups using analysis of covariance, additionally adjusting for baseline PaO_2_/FiO_2_ ratio value. For further detail on the statistical methods and sample size determination, see supplementary material.

## Results

The first patient in Part 1 was screened on 25 January 2021 and the last patient completed on 26 July 2021. The first patient in Part 2 was screened on 22 November 2021 and the study was closed in July 2022 due to an insufficient rate of patient recruitment. See supplementary material for Part 1 results.

### Patient disposition

**Fig. 1:**
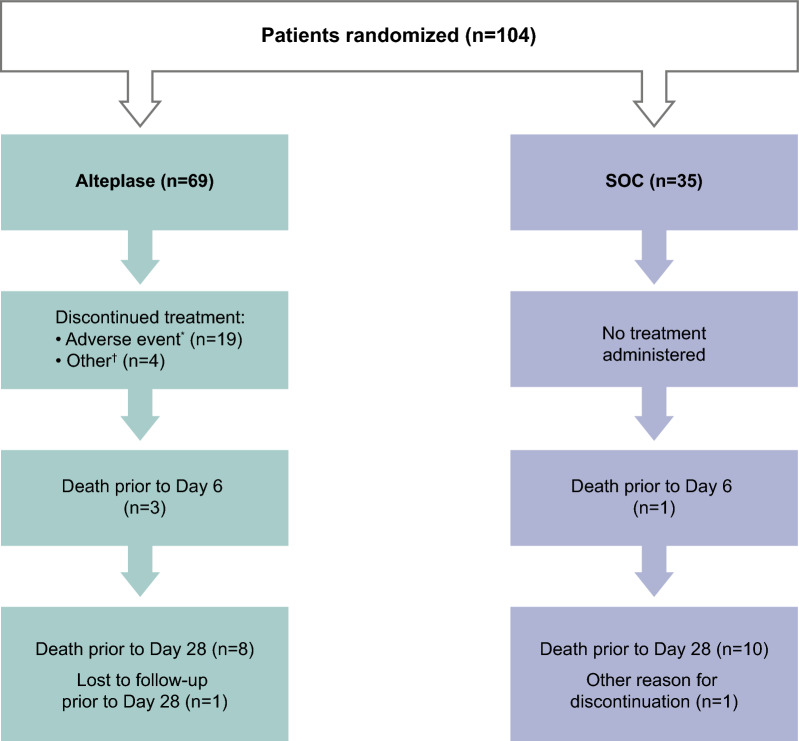
Patient flow diagram (pooled data). All randomized patients were treated and included in the full analysis set. *A full list of adverse events leading to discontinuation is provided in supplementary material (Table S12). ^†^Other patients discontinued due to loss of follow-up due to transfer out of ICU (n = 1), improvement in PaO_2_/FiO_2_ ratio of > 300 (n = 1), low fibrinogen level (n = 1), and mild bleeding (n = 1). *FiO*_*2*_ fractional inspired oxygen, *ICU* intensive care unit, *PaO*_*2*_ arterial oxygen partial pressure), *SOC* standard of care

Overall, 104 patients were randomized, of whom 69 patients were treated with alteplase plus SOC and 35 patients with SOC alone (Fig. 1).

### Baseline characteristics

Baseline characteristics were similar between the alteplase and SOC groups (Table 1). The mean age was 61.5 years, 65.4% of the patients were on non-invasive ventilation or high-flow oxygen (WHO Clinical Progression Scale score = 6; see Table S2), and the mean time since diagnosis of COVID-19 was 9.1 days. Relative to the SOC group, the alteplase group had a higher proportion of male patients (75% vs. 57%) and lower baseline median PaO_2_/FiO_2_ ratio (118.0 mmHg vs. 125.8 mmHg) (Table 1). For baseline characteristics by subgroup, see Tables S6 and S9.

**Table 1:** Baseline characteristics

	Alteplase pooled	SOC	Total
Number of patients, n (%)	69	35	104
Age (years), mean (SD)	61.5 (10.9)	61.4 (12.2)	61.5 (11.3)
Male, n (%)	52 (75.4)	20 (57.1)	72 (69.2)
Race*, n (%)			
White	43 (62.3)	18 (51.4)	61 (58.7)
Other	2 (2.9)	3 (8.6)	5 (4.8)
Not recorded	24 (34.8)	14 (40.0)	38 (36.5)
BMI (kg/m^2^), mean (SD)	30.5 (5.2)	29.8 (3.8)	30.2 (4.8)
Time since diagnosis (days), mean (SD)	9.3 (6.8)	8.7 (5.2)	9.1 (6.3)
Smoking status, n (%)			
Never	49 (71.0)	24 (68.6)	73 (70.2)
Former	17 (24.6)	8 (22.9)	25 (24.0)
Current	2 (2.9)	0	2 (1.9)
Missing	1 (1.4)	3 (8.6)	4 (3.8)
SOFA total score, mean (SD)	4.7 (2.3)	4.6 (2.2)	4.7 (2.3)
Baseline PaO_2_/FiO_2_ ratio (worst daily value), median (Q1, Q3)	118.0 (103.1, 160.0)	125.8 (105.3, 166.5)	122.0 (103.7, 164.3)
WHO scale, n (%)			
Score of 6	49 (71.0)	24 (68.6)	73 (70.2)
Score of 7	3 (4.3)	6 (17.1)	9 (8.7)
Score of 8	10 (14.5)	1 (2.9)	11 (10.6)
Score of 9	7 (10.1)	4 (11.4)	11 (10.6)
Supportive care type, n (%)			
Oxygen by mask or nasal prongs	1 (1.4)	1 (2.9)	2 (1.9)
Oxygen by high-flow mask or nasal cannula	24 (34.8)	7 (20.0)	31 (29.8)
Non-invasive ventilation	23 (33.3)	14 (40.0)	37 (35.6)
Invasive mechanical ventilation	20 (29.0)	9 (25.7)	29 (27.9)
Missing	1 (1.4)	4 (11.4)	5 (4.8)
Concomitant therapy			
Dexamethasone	52 (75.4)	28 (80.0)	80 (76.9)
Tocilizumab (IL-6 inhibitor)	6 (8.7)	4 (11.4)	10 (9.6)
D-dimer status, n (%)			
≥ ULN to < 3-fold ULN	2 (2.9)	1 (2.9)	3 (2.9)
3 to < 5-fold ULN	29 (42.0)	11 (31.4)	40 (38.5)
≥ 5-fold ULN	37 (53.6)	23 (65.7)	60 (57.7)
Missing	1 (1.4)	0	1 (1.0)

Alteplase pooled: 0.3 or 0.6 mg/kg over 2 h, followed by daily long-term (12-h) infusion of 0.02 or 0.04 mg/kg/h over 5 days (added to SOC)

*BMI* body mass index, *FiO*_*2*_ fraction of inspired oxygen, *IL* interleukin, *PaO*_*2*_ partial pressure of oxygen, *SD* standard deviation, *SOC* standard of care, *SOFA* Sequential (sepsis-related) Organ Failure Assessment, *ULN* upper limit of normal, *WHO* World Health Organization

^*^Data on race were not recorded in France (the largest recruiter of patients in this trial)

### Primary endpoint

**Fig. 2:**
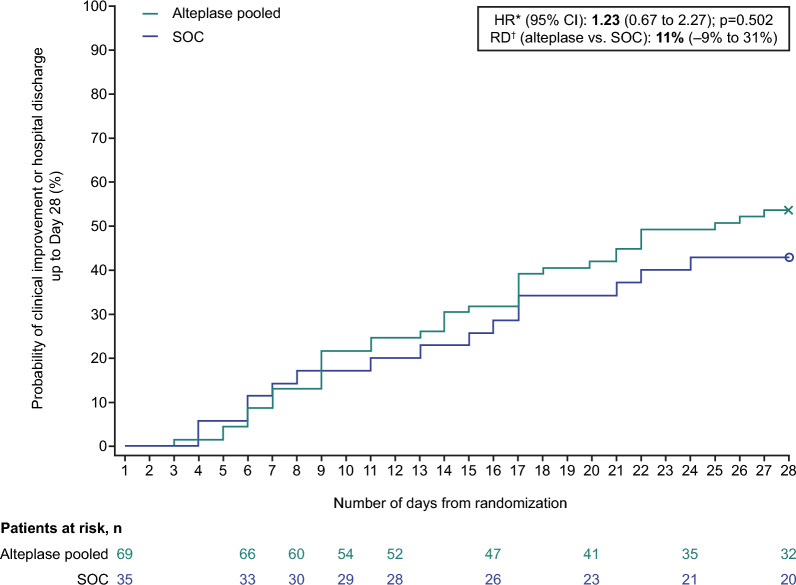
Time to clinical improvement up to Day 28. Alteplase pooled: 0.3 or 0.6 mg/kg over 2 h, followed by daily long-term (12-h) infusion of 0.02 or 0.04 mg/kg/h over 5 days (added to SOC). The symbols on the graphs represent censoring. *HR adjusted for treatment, baseline D-dimer status, age, baseline ventilation, and Part 1 or 2 of the study. ^†^RD corresponds to Day 28. *HR* hazard ratio, *RD* risk difference, *SOC* standard of care

The median time to clinical improvement up to Day 28 was 25 days in the alteplase group and > 28 days (median not reached) in the SOC group (Fig. 2; Table 2). The adjusted HR for alteplase versus SOC for the primary endpoint was 1.23 (95% CI 0.67 to 2.27; p = 0.502) (Table 2), with event rates within 28 days of 37/69 patients (54%) in the alteplase group and 15/35 patients (43%) in the SOC group (risk difference: + 11% [95% CI − 9% to 31%], Table 2; Fig. 2; Figure S2). In patients receiving high-dose alteplase, the risk difference was + 16% (− 5% to 38%) versus − 3% (− 30% to 24%) in those receiving low-dose alteplase (supplementary material, Table S7 and Figures S3 and S4). In patients who were not on invasive ventilation, the risk difference was + 27% (2% to 52%) (supplementary material, Table S10, Figures S5 and S6). Figure S7 shows a breakdown of WHO Clinical Progression Scale status at Day 28 in the high-dose alteplase group versus the SOC group (patients not on invasive ventilation only).

**Table 2:** Primary and secondary endpoints

	Alteplase pooled N = 69	SOC alone N = 35	p-value
Primary endpoint			
Time to clinical improvement* up to Day 28			
Median days to clinical improvement (95% CI)	25 (17 to NR)	NR^†^	
Patients with event, n (%)	37 (54)	15 (43)	
HR vs. SOC (95% CI)			
Unadjusted	1.31 (0.72 to 2.38)	–	0.383
Adjusted^‡^	1.23 (0.67 to 2.27)	–	0.502
Risk difference vs. SOC (95% CI)	11% (− 9 to 31)		
Key secondary endpoints			
Treatment failure (all-cause mortality or mechanical ventilation) up to Day 28			
Patients with event, n (%)	27 (39)	17 (49)	
Risk difference vs. SOC (95% CI)			
Unadjusted	− 9% (− 30 to 11)	–	0.359
Adjusted^‡^	− 8% (− 27 to 12)	–	0.448
All-cause mortality up to Day 28			
Patients with event, n (%)	8 (12)	10 (29)	
HR vs. SOC (95% CI)			
Unadjusted	0.39 (0.15 to 0.99)	–	0.048
Adjusted^‡^	0.42 (0.16 to 1.10)	–	0.077
Risk difference vs. SOC (95% CI)			
Unadjusted	− 17% (− 34 to 0)	–	0.047
Adjusted^‡^	− 16% (− 31 to 1)	–	0.058
Other secondary endpoints			
PaO_2_/FiO_2_ ratio (worst daily value) change from baseline up to Day 6			
Mean ± SD, mmHg	30.3 ± 84.3	− 11.7 ± 59.1	
Mean difference vs. SOC (95% CI)			
Unadjusted	37 (5 to 69)	–	0.023
Adjusted^§^	30 (0 to 59)	–	0.052
Length of hospital stay up to Day 28^¶^			
Mean ± SD, days	23.0 ± 7.3	24.4 ± 5.9	
Mean difference vs. SOC (95% CI)			
Unadjusted	− 1.4 (− 4 to 1)	–	0.331
Adjusted^‡^	− 1.0 (− 4 to 2)	–	0.449
Number of oxygen-free days up to Day 28^¶^			
Mean ± SD, days	6.7 ± 8.4	4.5 ± 7.1	
Mean difference vs. SOC (95% CI)			
Unadjusted	2.2 (− 1 to 6)	–	0.188
Adjusted^‡^	1.7 (− 2 to 5)	–	0.291
Further endpoint			
All-cause mortality up to Day 90			
Patients with event, n (%)	17 (25)	14 (40)	
Risk difference vs. SOC (95% CI)			
Unadjusted	− 15% (− 35 to 4)	–	0.116
Adjusted^‡^	− 14% (− 33 to 4)	–	0.134
Safety endpoint			
Major bleeding event up to Day 6			
Patients with event, n (%)	9 (13)	0 (0)	
Risk difference vs. SOC (95% CI)	13% (1 to 23)	–	< 0.05

Alteplase pooled: 0.3 or 0.6 mg/kg over 2 h, followed by daily long-term (12-h) infusion of 0.02 or 0.04 mg/kg/h over 5 days (added to SOC)

*CI* confidence interval, *FiO*_*2*_ fraction of inspired oxygen, *HR* hazard ratio, *NA* not available, *NR* not reached, *PaO*_*2*_ partial pressure of oxygen, *SD* standard deviation, *SOC* standard of care, *WHO* World Health Organization

*Improvement of ≥2 points on the 11-point WHO Clinical Progression Scale, or discharge from the hospital, whichever came first

^†^WHO Clinical Progression score was recorded up to 28 days, and by Day 28, less than 50% of SOC patients had a clinical improvement. Therefore, median (50%) was not reached by Day 28

^‡^Adjusted for treatment, baseline D-dimer status, age, baseline ventilation status, and Part 1 or 2 of the study

^§^Adjusted for treatment, baseline PaO_2_/FiO_2_ ratio, baseline D-dimer status, age, baseline ventilation status, and Part 1 or 2 of the study

^¶^In the event of death, the length of a patient’s hospital stay was automatically recorded as 28 days, and the number of oxygen-free days was 0

### Secondary endpoints

The adjusted risk of treatment failure (all-cause mortality or mechanical ventilation [Day 28]) was similar in the alteplase and SOC groups (risk difference: − 8% [95% CI − 27% to 12%]; p = 0.448) (Table 2).

**Fig. 3:**
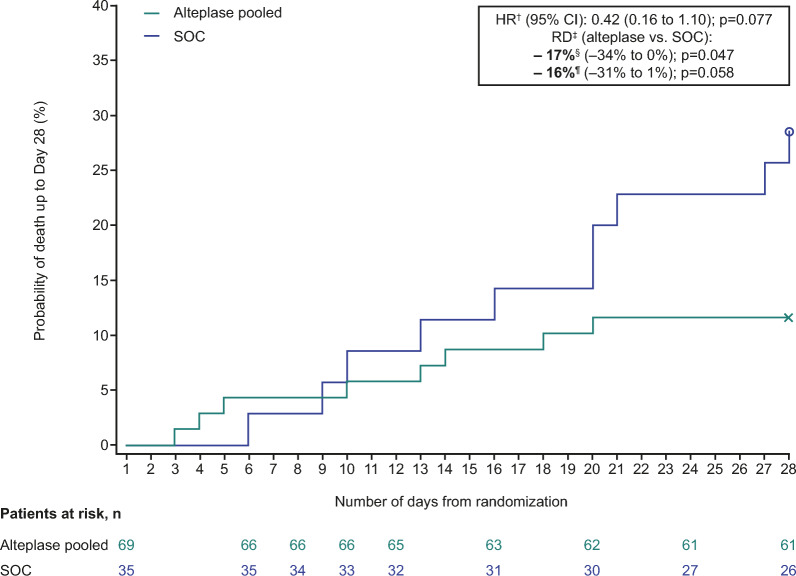
Time to all-cause mortality up to Day 28*. Alteplase pooled: 0.3 or 0.6 mg/kg over 2 h, followed by daily long-term (12-h) infusion of 0.02 or 0.04 mg/kg/h over 5 days (added to SOC). *Time to all-cause mortality was not a predefined endpoint. Kaplan–Meier analysis was carried out retrospectively, based upon the results of the key secondary endpoint (all-cause mortality). ^†^Cox proportional hazard model adjusted for fixed effects for treatment, baseline D-dimer status, age, baseline ventilation, and Part 1 or 2 of the study. ^‡^RD corresponds to Day 28. ^§^Unadjusted RD. ^¶^Adjusted RD based upon the binary endpoint, adjusted for treatment, baseline D-dimer status, age, baseline ventilation, and Part 1 or 2 of the study. *CI* confidence interval, *HR* hazard ratio, *RD* risk difference, *SOC* standard of care

Overall, patients in the alteplase group had a significantly lower risk of all-cause mortality (Day 28) compared with those in the SOC group (patients with events, 8/69 [12%] vs. 10/35 [29%]; unadjusted risk difference: − 17% [95% CI − 34% to 0%]; p = 0.047) (Table 2; Fig. 3). However, after adjustment, this finding was not significant (risk difference: − 16% [95% CI − 31% to 1%; p = 0.058]) (Table 2). Post hoc time-to-event analyses of all-cause mortality were consistent with the overall reduction in mortality risk in the alteplase group (unadjusted HR: 0.39 [95% CI 0.15 to 0.99], p = 0.048; adjusted HR: 0.42 [95% CI 0.16 to 1.10], p = 0.077) (Table 2). All-cause mortality up to Day 90 was 17/69 (25%) in the alteplase group versus 14/35 (40%) in the SOC group (unadjusted risk difference: − 15% [95% CI − 35% to 4%]; p = 0.116), with similar findings after adjustment (Table 2; for further information, see supplementary material). Risk differences for both treatment failure (Day 28) and all-cause mortality (Day 28 and Day 90) were similar regardless of dose group (see Table S7).

Mean change from baseline in PaO_2_/FiO_2_ ratio (mean ± standard deviation) at Day 6 was higher in the alteplase group (30.3 ± 84 mmHg) than in the SOC group (− 11.7 ± 59 mmHg). The unadjusted mean difference was + 37 mmHg (95% CI 5 to 69; p = 0.023) for alteplase versus SOC (Table 2); this was clinically relevant for the high dose (+ 49 mmHg) but not for the low dose (+ 9 mmHg) (see Table S7). After adjustment, the mean difference was + 30 mmHg (95% CI 0 to 59; p = 0.052). In patients who were not on invasive ventilation, the unadjusted mean difference for alteplase versus SOC was + 78 mmHg (supplementary material, Table S10).

For other secondary endpoints (length of hospital stay and number of oxygen-free days), differences between alteplase and SOC were not statistically significant, apart from the number of oxygen-free days in patients who were not on invasive ventilation (Table 2; see Tables S7 and S10). Sensitivity analyses of key secondary endpoints, adjusted for a combination of variables, showed results consistent with the main analyses (see Table S13).

### Safety

The frequency of adverse events (AEs) and serious AEs (SAEs) was similar in the alteplase and SOC groups (Table 3). SAEs occurring at a frequency of > 5% in the alteplase group were epistaxis (12%), PE (12%), pneumonia (9%) and oral hemorrhage (6%). In the SOC group, SAEs occurring at a frequency of > 5% were respiratory failure (20%), septic shock (14%), PE (6%), deep vein thrombosis (6%) and multi-organ dysfunction syndrome (6%).

**Table 3:** Adverse events and bleeding

	Alteplase both doses (N = 69)	SOC (N = 35)
Any AEs	60 (87.0)	30 (85.7)
Severe AEs	24 (34.8)	16 (45.7)
Investigator-defined, drug-related AEs	38 (55.1)	NA
AEs leading to discontinuation	18 (26.1)	NA
Bleeding events		
Treatment-emergent * bleeding	35 (50.7)	4 (11.4)
Blood transfusion needed^†^	3 (4.3)	1 (2.9)
Major bleeding	10 (14.5)	1 (2.9)
Fatal^**‡**^	0 (0)	0 (0)
Non-major bleeding	28 (40.6)	3 (8.6)
Fatal^**‡**^	0 (0)	0 (0)
Serious AEs	34 (49.3)	18 (51.4)
Fatal	7 (10.1)	6 (17.1)
Life threatening	9 (13.0)	8 (22.9)
Required or prolonged hospitalization	12 (17.4)	9 (25.7)
Other significant AEs according to ICH E3^§^	7 (10.1)	NA

Data represent number of patients, n (%)

*AE* adverse event, *ICH* International Council for Harmonization of Technical Requirements for Pharmaceuticals for Human Use, *NA* not applicable, *SOC* standard of care

^*^Alteplase patients: Start point = date and time of first administration of alteplase; end point = latest of start point + 288 h or last administration of alteplase + 168 h. SOC patients: Start point = date and time of randomization. End point = start point + 288 h

^**†**^Whole blood cell or packed red blood cell transfusion

^‡^Fatal bleeds were defined as a bleeding event that the investigator determined was the primary cause of death or contributed directly to death

^§^Other significant AEs are non-serious AEs leading to treatment discontinuation

Treatment-emergent bleeding occurred in 50.7% of patients in the alteplase group and in 11.4% of patients in the SOC group. Major bleeding occurred in 14.5% of patients on alteplase compared with 2.9% of patients on SOC (n = 1 in the SOC group reported intracranial hemorrhage). However, no bleeding was fatal. The risk of MBEs within the first 6 days of treatment was 13% higher in the alteplase versus SOC group (risk difference vs. SOC, p < 0.05), occurring in nine patients on alteplase and no patients receiving SOC (Table 2 and Table S14). The risk difference for MBEs was greater for high-dose alteplase (+ 16%) compared with low-dose alteplase (+ 5%) (see Table S8), and similar to the pooled cohort in patients who were not on invasive ventilation (+ 12%) (see Table S11). Four patients received a blood transfusion (one patient on low-dose alteplase, two on high-dose alteplase and one receiving SOC, see Table 3 and Table S8). For AEs and bleeding stratified by alteplase dose and for patients not receiving invasive ventilation, see Tables S8 and S11, respectively.

In the pooled alteplase group, 18 patients (26.1%) had AEs that led to treatment discontinuation (Table 3). The most frequent of these were epistaxis (n = 7), hematoma (n = 3), catheter site hemorrhage (n = 3), hematuria (n = 2), oral hemorrhage (n = 2), pharyngeal hemorrhage (n = 2) and PE (n = 2) (for further details, see supplementary material and Table S12).

## Discussion

In this multinational Phase IIb/III trial (TRISTARDS), patients with COVID-19 and severe hypoxemic respiratory failure who were randomized to receive the thrombolytic drug alteplase in addition to SOC had no difference in the primary endpoint—time to clinical improvement at Day 28—compared with patients receiving SOC alone. However, we observed numerical differences in survival and PaO_2_/FiO_2_ ratio in the alteplase group, meriting further investigation of whether thrombolysis (alongside prophylactic anticoagulation) might play a role in preventing clinical deterioration of patients with severe hypoxemic respiratory failure associated with COVID-19.

Our findings, in a population of patients who were mainly (90%) treated in an intensive care unit but were mostly not intubated (70%), show numerical differences in survival, which were most pronounced in patients on non-invasive ventilation (as shown by the subgroup analysis of patients with a WHO score of 6). A meta-analysis of high-quality randomized controlled trials has previously suggested that in hospitalized but not critically ill patients with COVID-19, full-dose (therapeutic) heparin-based anticoagulation reduces the number of thrombotic events and is associated with lower mortality [29]. In contrast, in previous studies of critically ill patients with COVID-19, therapeutic-dose or high-dose prophylactic anticoagulant therapy did not increase the probability of survival to hospital discharge [30] or improve mortality and time to clinical improvement [31] compared with standard-dose prophylactic anticoagulation.

At the time of study conduct, treatment options for patients hospitalized with COVID-19 and requiring oxygen and/or ventilation support were evolving. In addition, guidelines recommended prophylactic anticoagulation for all patients with COVID-19 in the intensive care unit [32, 33]. Based on several small-scale exploratory studies and patient case series, it was hypothesized that critically ill patients with COVID-19 and hypercoagulation (as defined by increased D-dimer levels) might benefit from thrombolytic therapy with alteplase, in addition to prophylactic anticoagulant therapy with low-molecular-weight heparins or unfractionated heparin, early in the disease course [16–19, 26, 34]. Notably, the STARS and MUST COVID trials both showed that, in patients with severe COVID-19-induced ARDS who were on mechanical ventilation, alteplase was safe and improved PaO_2_/FiO_2_ ratio [35, 36].

The study completion rate in TRISTARDS was high, with only one of the 69 patients in the alteplase group lost to follow-up. Pooling of patient data from Parts 1 and 2 showed a marginally significant reduction in all-cause mortality and improved PaO_2_/FiO_2_ ratio in patients receiving alteplase. Improvement in PaO_2_/FiO_2_ ratio up to Day 6 in the alteplase group may have represented the first sign of clinical benefit, translating into greater survival from Day 10 onwards, i.e. the point at which the alteplase and SOC groups diverge within the KM plots. For all efficacy endpoints, the observed treatment differences were most pronounced in patients receiving high-dose alteplase and in those who were not on invasive ventilation, suggesting that administration of alteplase during an early therapeutic window may prevent deterioration more effectively than in more severely ill patients who are already mechanically ventilated, i.e. have more severe hypoxemia.

Although major bleedings were more common in the pooled alteplase group compared with the SOC group (14.5% vs. 2.9%), none of these were fatal. For comparison, in studies of therapeutic-dose or high-dose prophylactic anticoagulant therapy in a severe COVID setting, the rate of major or severe bleeding was lower, i.e. in the range of 2.1–3.8% compared with 0.5–2.3% with standard therapy [30, 31, 37]. Of note, in TRISTARDS, there was only one case of intracranial hemorrhage reported in the SOC group and none in the alteplase group. Furthermore, the risk of major bleeding associated with alteplase should be interpreted in the context of the in-hospital mortality rate for patients with hypoxemic respiratory failure and a PaO_2_/FiO_2_ ratio of 100–300 (i.e. the patient population in TRISTARDS), which is estimated at approximately 35–46% based on a large-scale analysis of intensive care unit outcomes in 50 countries [38]. Moreover, the overall number of AEs and SAEs was similar in both groups, although higher rates of investigator-defined drug-related AEs and AEs leading to discontinuation of alteplase were observed with high-dose versus low-dose alteplase.

The study had several limitations, including the low power of the study. As the intensity of the pandemic decreased, the most common clinical presentation of COVID-19 evolved into a milder form of illness, resulting in very few patients suffering from severe hypoxemic respiratory failure. As a result, Part 2 of the study was closed early, due to insufficient patient recruitment (as expected, patients in Part 2 had less severe hypoxemic respiratory failure compared with Part 1). Hence, none of the efficacy results can be considered conclusive (especially in the IMV cohort), due to the low sample size. Furthermore, pooling of data from high- and low-dose alteplase groups in the main analysis makes it hard to draw any conclusion and is the main limitation of the study.

Although all necessary adjustments were made in accordance with established guidelines and best practices, potential confounding due to differences in baseline characteristics between pooled subgroups is possible, for example, due to an imbalance in disease severity between the groups. However, the results of adjusted and unadjusted analyses had the same magnitude and direction as the primary analyses, despite the relatively small cohort size. In addition, as the study was open-label, it is not possible to exclude the possibility that some therapeutic interventions may have been performed differently between the groups. For example, prophylactic anticoagulant therapy was not standardized and was administered at the physician’s discretion. However, the available evidence prior to study conduct supported anticoagulation at prophylactic or intermediate dosages, and the classification of anticoagulant regimens by intensity was based on the American Society of Hematology 2021 guidelines [33]. In addition, anticoagulant therapy was monitored in this trial using activated partial thromboplastin time, but this may have influenced the proportion of bleeding in critically ill patients with COVID-19, either in the treatment or control group; hence, monitoring using anti-Xa levels may be preferable in future studies [39].

A further limitation is that the primary outcome was partially subjective, since the WHO scale comprises steps heavily driven by physician decisions such as extubation and non-invasive ventilation. Also, lack of treatment blinding is another potential limitation, though this was not considered reasonable or feasible given the design and clinical context of the study. Lastly, at the time of the study, the SOC for treatment of severe COVID-19 did not yet include interleukin-6 (IL-6) inhibitors such as tocilizumab, and thus baseline use of this treatment in our study was low (9–11% of patients). It is possible that outcomes may have differed with higher baseline use of IL-6 inhibitors, though previous studies of patients hospitalized with COVID-19 (regardless of severity) have not shown any survival benefit of tocilizumab in addition to SOC [40, 41].

Pulmonary microthrombi and associated ARDS have been linked to fatal outcomes in pandemics caused by bacterial and viral infections since 1918 [1–3]. Along with numerical differences in survival and PaO_2_/FiO_2_ ratio in patients with COVID-19, alteplase may also benefit patients with severe hypoxemic failure caused by other infections, meriting further investigation. Exploratory findings from this study will help to inform future trial design and sample size calculation. Given that there has been a severe coronavirus respiratory pandemic every 10 years for the last 3 decades (SARS-CoV in 2003, MERS-CoV in 2012 and COVID-19 in 2019) [1], further evaluation of pharmacologic agents including thrombolytics and anticoagulants is warranted and might improve respiratory outcomes in patients with severe disease.

## Supplementary Information

Supplementary Material 1.

## Data Availability

The datasets used and/or analyzed during the current study are available from the study sponsor, Boehringer Ingelheim, on reasonable request. To ensure independent interpretation of clinical study results and enable authors to fulfill their role and obligations under the ICMJE criteria, Boehringer Ingelheim grants all external authors access to relevant clinical study data. In adherence with the Boehringer Ingelheim Policy on Transparency and Publication of Clinical Study Data, scientific and medical researchers can request access to clinical study data, typically, one year after the approval has been granted by major Regulatory Authorities or after termination of the development program. Researchers should use the https://vivli.org/ link to request access to study data and visit https://www.mystudywindow.com/msw/datasharing for further information.

## Abbreviations

AE: Adverse event
ARDS: Acute respiratory distress syndrome
CI: Confidence interval
COVID-19: Coronavirus disease 2019
FiO_2_: Fractional inspired oxygen
HR: Hazard ratio
IMV: Invasive mechanical ventilation
IL: Interleukin
i.v.: Intravenous
KM: Kaplan–Meier
MBE: Major bleeding event
MERS-CoV: Middle East respiratory syndrome
PaO_2_: Arterial oxygen partial pressure
PE: Pulmonary embolism
SAE: Serious adverse event
SARS-CoV-2: Severe acute respiratory syndrome coronavirus 2
SOC: Standard of care
TRISTARDS: ThRombolysIS Therapy for ARDS
ULN: Upper limit of normal
WHO: World Health Organization
