# Supplementary material for: Alteplase in COVID-19 severe hypoxemic respiratory failure: the TRISTARDS multicenter randomized trial
Source: Ann Intensive Care. 2024 Nov 10;14:170. doi: 10.1186/s13613-024-01386-z (PMC11551089; doi:10.1186/s13613-024-01386-z)
Supplement: Supplementary file 1 — Supplementary Material 1. [file 13613_2024_1386_MOESM1_ESM.docx]

**Supplementary material**

**Alteplase in COVID-19 severe hypoxemic respiratory failure: the TRISTARDS multicenter randomized trial**

Giovanni Landoni, MD^1,2*^, Pratima Chowdary, MD^3*^, Ferhat Meziani, MD, PhD^4^, Jacques Creteur^5^, Nicolas De Schryver, MD^6^, Johann Motsch, MD^7^, Ingrid Henrichmoeller, MD^8,9^, Alain Pagès, MD^8^, Nuala Peter, MSc^10^, Thierry Danays, MD^11^ Markus A. Weigand, MD^7^; for the TRISTARDS investigators

^1^Department of Anesthesia and Intensive Care, IRCCS San Raffaele Scientific Institute, Milan, Italy

^2^School of Medicine, Vita-Salute San Raffaele University, Milan, Italy

^3^Katharine Dormandy Haemophilia and Thrombosis Centre, Royal Free Hospital, London, United Kingdom

^4^Université de Strasbourg (UNISTRA), Faculté de Médecine; Hôpitaux Universitaires de Strasbourg, Nouvel Hôpital Civil, Service de Médecine Intensive-Réanimation, Strasbourg, France

^5^Department of Intensive Care, ULB Hôpital Erasme, Brussels, Belgium

^6^Intensive Care Unit, Clinique St-Pierre, Ottignies, Belgium

^7^Department of Anesthesiology, Heidelberg University Hospital, Heidelberg, Germany

^8^Therapeutic Area Cardiovascular Medicine, Boehringer Ingelheim International GmbH, Ingelheim, Germany

^9^Fifth Department of Medicine, Faculty of Medicine Mannheim, University of Heidelberg, Mannheim, Germany

^10^Statistician, Boehringer Ingelheim, Biberach an der Riss, Germany

^11^TDC, Aix en Provence, France

^*^Joint first authors.

**Table of contents**

[Methods 5](#_Toc178923638)

[Table S1. Patients by country and TRISTARDS trial collaborators (Parts 1 and 2 – FAS) 5](#_Toc178923639)

[Table S2. WHO Clinical Progression Scale [1] 13](#_Toc178923640)

[Data monitoring committee 14](#_Toc178923641)

[Rationale for dose selection 14](#_Toc178923642)

[Measures implemented to prevent clot formation 15](#_Toc178923643)

[Treatments included in SOC 16](#_Toc178923644)

[Inclusion/exclusion criteria 16](#_Toc178923645)

[Statistical analyses 17](#_Toc178923646)

[Statistical analyses: Adjustments 20](#_Toc178923647)

[Sensitivity analyses of key secondary endpoints 22](#_Toc178923648)

[Definition of major bleeding 22](#_Toc178923649)

[Figure S1. Study design 23](#_Toc178923650)

[Table S3. FiO_2_ ranges for common oxygen delivery devices 24](#_Toc178923651)

[Table S4. Values to derive PaO_2_/FiO_2_ ratio from SpO_2_ 25](#_Toc178923652)

[Table S5. Summary of cohorts used in statistical analyses 27](#_Toc178923653)

[Results 28](#_Toc178923654)

[Part 1 results 28](#_Toc178923655)

[Primary endpoint 28](#_Toc178923656)

[Secondary endpoints 28](#_Toc178923657)

[AEs 29](#_Toc178923658)

[Pooled results (Parts 1&2) 29](#_Toc178923659)

[All-cause mortality at Day 90 29](#_Toc178923660)

[Figure S2. Time to clinical improvement or hospital discharge up to Day 28 (Parts 1 and 2, alteplase pooled vs. standard of care) 30](#_Toc178923661)

[Supplementary analysis 1 (Subgroup 1): Patients stratified according to alteplase dose (Parts 1 and 2 pooled) 31](#_Toc178923662)

[Figure S3. Patient population: all patients (Parts 1 and 2), stratified by dose 31](#_Toc178923663)

[Figure S4. Time to clinical improvement up to Day 28: all patients (Parts 1 and 2 vs SOC), stratified by dose 32](#_Toc178923664)

[Table S6. Baseline characteristics: all patients (Parts 1 and 2), stratified by dose 33](#_Toc178923665)

[Table S7. Primary, secondary and safety endpoints: all patients (Parts 1 and 2), stratified by dose 35](#_Toc178923666)

[Table S8. TEAEs* and bleeding: all patients (Parts 1 and 2), stratified by dose 39](#_Toc178923667)

[Supplementary analysis 2 (Subgroup 2): Patients not on invasive ventilation (Parts 1 and 2 pooled) 41](#_Toc178923668)

[Figure S5. Patient population: patients not on invasive ventilation (Parts 1 and 2)* 41](#_Toc178923669)

[Figure S6. Time to clinical improvement or hospital discharge up to Day 28: patients not on invasive ventilation (Parts 1* and 2 vs. SOC, stratified by study part) 42](#_Toc178923670)

[Figure S7. WHO Clinical Progression Scale status at Day 28: patients not on invasive ventilation (Parts 1 and 2 pooled, high-dose alteplase vs. standard of care) 43](#_Toc178923671)

[Table S9. Baseline characteristics: patients not on invasive ventilation (Parts 1 and 2) 44](#_Toc178923672)

[Table S10. Primary, secondary and safety endpoints: patients not on invasive ventilation (Parts 1 and 2) 46](#_Toc178923673)

[Table S11. TEAEs* and bleeding: patients not on invasive ventilation (Parts 1 and 2) 50](#_Toc178923674)

[Table S12. TEAEs* leading to discontinuation of alteplase 52](#_Toc178923675)

[Table S13. Sensitivity analyses of key secondary endpoints: all patients (Parts 1 and 2) 54](#_Toc178923676)

[Table S14. Major bleeding events up to Day 6 in patients receiving alteplase^*^ 56](#_Toc178923677)

[Supplemental references 57](#_Toc178923678)

# Methods

## Table S1. Patients by country and TRISTARDS trial collaborators (Parts 1 and 2 – FAS)

| **Austria (10 patients)** |
| --- |
| **Dr. Alexander Zoufaly, MD^*^** |
| Dr. Tamara Seitz, MD |
| Dr. Erich Pawelka, MD |
| Dr. Stephanie Neuhold, MD |
| Dr. Wolfgang Höpler, MD |
| Dr. Benedikt Rumpf, MD |
| Dr. David Totschnig, MD |
| **Prof. Rudolf Likar, MD^*^** |
| Dr. Markus Koestenverger, MD |
| Dr. Stefan Neuwersch-Sommeregger, MD |
| **Belgium (20 patients)** |
| **Prof. Jacques Creteur, MD PhD^*^** |
| Dr. Amedee Ego, MD |
| Dr. Anthony Moreau, MD |
| Dr. David Grimaldi, MD |
| Dr. Filippo Annoni, MD |
| Dr. Julie Gorham, MD |
| Dr. Katarina Halenarova, MD |
| Dr. Zoe Pletschette, MD |
| Dr. Alexandre Brasseur, MD |
| Dr. Fabio Taccone, MD |
| Dr. Leda Nobile, MD |
| Dr. Olivier Lheureux, MD |
| Dr. Morgane Snacken, MD |
| Dr. Charles Dehout, MD |
| **Dr. Nicolas De Schryver^*^** |
| Dr. Marco Vinetti |
| Dr. Nicolas Serck |
| Dr. Thierry Dugernier |
| **Dr. Nathalie Layios^*^** |
| Dr. Gilles Parzibut |
| **Brazil (1 patient)** |
| **Dr. Rodrigo Boldo^*^** |
| Dr. Vanessa Santos dos Santos |
| Dr. Cristine Erdmann Nunes |
| **France (38 patients)** |
| **Prof. Jean-Luc DIEHL^*^** |
| N. Aissaoui, MD |
| J. Augy, MD |
| E. Guerot, MD |
| C. Hauw-Berlemont, MD |
| B. Hermann, MD |
| N. Peron, MD |
| F. Santi, MD |
| J. Langlais, MD |
| A. Troger, MD |
| *K Chekhrit* |
| **Prof. Julien POISSY^*^** |
| M Caplan, MD |
| A El Kalioubie, MD |
| R Favory, MD |
| A Gaudet, MD |
| J Goutay, MD |
| S Preau, MD |
| A Rouze, MD |
| *Laure Mariller* |
| **Prof. Jean-Paul MIRA^*^** |
| Z Ait Hamou, MD |
| S Ben Ghanem, MD |
| M Bertrix, MD |
| J Charpentier, MD |
| T Creutin, MD |
| M Jozwiak, MD |
| D Laghlam, MD |
| E Peju, MD |
| F Pene, MD |
| C Vigneron, MD |
| **Prof. Ferhat MEZIANI^*^** |
| J Demisselle, MD |
| J Helms, MD |
| L Jandeaux, MD |
| C Kummerlen, MD |
| H Merdji, MD |
| A Monnier, MD |
| H Rahmani, MD |
| A Studer, MD |
| S Cunat, MD |
| *Ouafa Hakkari* |
| **Prof. Xavier MONNET^*^** |
| I Adda, MD |
| N Anguel, MD |
| S Ayed, MD |
| Q Fosse, MD |
| L Guerin, MD |
| D Osman, MD |
| A Pavot, MD |
| T Pham, MD |
| C Carpentier, MD |
| P Denormandie, MD |
| C Lai, MD |
| *Alain Fourreau* |
| **Prof. Mehran MONCHI^*^** |
| O Ellrodt, MD |
| S Jochmans, MD |
| S Mazerand, MD |
| N Rolin, MD |
| J Serbource-Goguel, MD |
| P Soulier, MD |
| O Sy, MD |
| *Nourdine Benane* |
| **Prof. Bruno Mourvillier^*^** |
| J Cousson, MD |
| A Goury, MD |
| O Passouant, MD |
| G Thery, MD |
| *Cédric Castex* |
| **Jean-Baptiste Lascarrou, MD^*^** |
| A Roquilly, Prof. |
| E Canet, MD |
| C Garret, MD |
| J Lemarie, MD |
| M Martin, MD |
| J Reignier, Prof. |
| A Seguin, MD |
| O Zambon, MD |
| P Lamouche Wilquin, MD |
| M Agbakou, MD |
| P Decamps, MD |
| L Desmedt, MD |
| G Blonz, MD |
| Y Hourmant, MD |
| N Grillot, MD |
| A Rouhani, MD |
| M Bouras, MD |
| P-J Mahe, MD |
| D Demeure Dit Latte, MD |
| A Bourdiol, MD |
| N Benkalfate, MD |
| M Carpentier, MD |
| F Guillotin, MD |
| S Benguerfi, MD |
| **Germany (9 patients)** |
| **Prof. Dr. Johann Motsch^*^** |
| Dr. Johannes Zimmermann |
| Karam Al Halabi |
| Dr. Marc Altvater |
| Dr. Sebastian Decker |
| Dr. Mascha Fiedler |
| Prof. Dr. Phillip Knebel |
| Barbara Maichle |
| Prof. Dr. Markus Weigand |
| **Prof. Dr. Tobias Welte^*^** |
| Dr. Nora Drick |
| Dr. Isabelle Pink, 02 Sep 2020 – 07 May 2021 |
| Dr. Julius Johannes Schmidt |
| **Prof. Dr. Sven Bercker^*^** |
| Dr. Philipp Simon |
| Dr. Falk Fichtner |
| Dr. Gunther Hempel |
| Peter Kliem |
| Dr. Karsten Kluba |
| Dr. Sven Laudi |
| Dr. Sarah Müller |
| Dr. Rene Oesemann |
| Michael Roedel |
| Dr. Stefan Schering |
| Sebastian Schulz |
| Dr. Christian Seeber |
| Hannah Ullmann |
| Svitlana Ziganshyna |
| Dr. Nora Jahn |
| Dr. Bastian Boerge |
| Dr. Maren Keller |
| **PD Dr. Michael Irlbeck^*^** |
| Dr. Sandra Frank |
| **Prof. Dr. Ursula Hoffmann^*^** |
| Aydin Huseynov |
| Dr. Simone Britsch |
| Dr. Gill Ishar-Singh |
| Dr. Claude Jabbour |
| **Dr. Sven Stieglitz^*^** |
| Dr. Jan-Erik Guelker |
| **Italy (6 patients)** |
| **Maurizio Cecconi, MD^*^** |
| Massimiliano Greco, MD |
| **Giacomo Monti, MD^*^** |
| Maria Luisa Azzolini, MD |
| Beatrice Righetti, MD |
| **Mexico (2 patients)** |
| **Dr. Francisco Marquez Diaz^*^** |
| Dr. Sofía Elizabeth Girón |
| Dr. Alejandra Aviles de La Cruz |
| Dr. Ana Elena Ramírez Ibarra |
| Dr. Paola Hernández Romo |
| Dr. Marián Serna García |
| Dr. Andrés García Castillo |
| **Netherlands (4 patients)** |
| **Dr. Peter Spronk, MD^*^** |
| Dr Marnix Kuindersma, MD |
| **Dr. Michiel Blans^*^** |
| Dr. Henk Van Leeuwen |
| **Dr. Marco Peters^*^** |
| Dr. Els Rengers |
| Dr. Oscar Hoiting |
| **Russia (6 patients)** |
| **Dr. Viktor Borisovich Filimonov, MD^*^** |
| Dr. Maria Peshenniokva, MD |
| Dr. Olga Kravchenko, MD |
| Dr. Yuri Karev, MD |
| Dr. Anastasiia Filimonova, MD |
| **Prof: Sergey Nikolaevich Avdeev^*^** |
| Dr. Svetlana Chikina |
| Dr. Tatiana Gneusheva |
| Dr. Zamira Merzhoeva |
| Dr. Galina Nekludova |
| **Dr. Denis Nikolaevich Protsenko, MD^*^** |
| Dr. Igor Tyurin, MD |
| Dr. Nikita Matyushkov, MD |
| Dr. Tatiana Valerievna V Lisun, MD |
| Dr. Aleksandr Boyarkov, MD |
| Dr. Svetlana Bobkova, MD |
| **MD Alexey Klinov^*^** |
| MD Dmitry Schukarev |
| MD Nikolay Smolin |
| **Spain (8 patients)** |
| **Dr. Ricard Ferrer^*^** |
| Dr. Xavier Nuvials |
| Dra. Sofia Contreras |
| Dr. Alejandro Cortés |
| Dra. Mariel Rojas Lora |
| **Dr. Rafael Sierra^*^** |
| Dr. Samer Alarbe |
| Dra. Ana Fernandez |
| Dr. Mario Contreras |
| Dra María Dolores Freire |
| Dr. Jaume Revuelto |
| Dr. Mikel Celaya |
| **Dra. Judith Marín^*^** |
| Dr. Francisco Parrilla |
| Dra. Purificación Perez |
| Dra. Rosana Muñoz |
| **Dr. Emilio Diaz^*^** |
| Dr. Cristina Mora |
| Dr. Candelaria de Haro |
| Dr Edgard Moglia |

All enrolled patients were randomized, treated, and included in the full analysis set.

**^*^**Principal investigators

FAS, full analysis set.

## Table S2. WHO Clinical Progression Scale [1]

| **Patient state** | **Descriptor** | **Score** |
| --- | --- | --- |
| Uninfected | Uninfected; no viral RNA detected | 0 |
| Ambulatory mild disease | Asymptomatic; viral RNA detected | 1 |
|  | Symptomatic; independent | 2 |
|  | Symptomatic; assistance needed | 3 |
| Hospitalized:  moderate disease | Hospitalized; no oxygen therapy* | 4 |
|  | Hospitalized; oxygen by mask or nasal prongs | 5 |
| Hospitalized:  severe diseases | Hospitalized; oxygen by NIV or high flow | 6 |
|  | Intubation and mechanical ventilation, PaO_2_/FiO_2_ ≥150 or SpO_2_/FiO_2_ ≥200 | 7 |
|  | Mechanical ventilation PaO_2_/FiO_2_ <150 (SpO_2_/FiO_2_ <200) or vasopressors | 8 |
|  | Mechanical ventilation PaO_2_/FiO_2_ <150 and vasopressors, dialysis, or ECMO | 9 |
| Dead | Dead | 10 |

*****If hospitalized for isolation only, record status as for ambulatory patient.

ECMO, extracorporeal membrane oxygenation. FiO_2_, fractional inspired oxygen. NIV, non-invasive ventilation. PaO_2_, arterial oxygen partial pressure. SpO_2_, oxygen saturation; WHO, World Health Organization.

## Data monitoring committee

An independent data monitoring committee (DMC) was established. Members of the DMC were independent of the study sponsor (Boehringer Ingelheim) and included physicians experienced in the treatment of the disease under investigation and/or thrombolytic treatments, as well as a statistician. The DMC evaluated safety and efficacy data, as well as the results of the interim analysis. The DMC received urgent significant safety events, including cases of intracranial hemorrhage and fatal bleeds for immediate evaluation. Regular DMC meetings were held at specified intervals.

The DMC could recommend continuation, modification or termination of the trial. DMC recommendations and the final Boehringer Ingelheim decision were reported to the appropriate regulatory or health authorities, institutional review boards/ethics committees and investigators as requested by local law.

## Rationale for dose selection

Dose selection was based upon the established safety profile of alteplase, as well as pharmacokinetic and pharmacodynamic modelling results, which demonstrated a dose-dependent decrease of fibrinogen levels during infusion and recovery of these levels during infusion intervals (Boehringer Ingelheim, data on file). As defined in the protocol, Part 1 was planned to randomize 60 patients, stratified by type of ventilation (patients not on invasive ventilation vs. patients on invasive mechanical ventilation [IMV]).

The concept of combining a moderate initial dose of alteplase (0.6 mg/kg) over 2 hours (e.g. a total dose of 42 mg for a patient with 70 kg body weight), followed by a constant rate infusion (CRI) of low-dose alteplase (0.04 mg/kg/h) over 12 hours, was expected to reduce the risk of alteplase-related bleeding events compared with higher doses of alteplase used in other indications (e.g. 100 mg for massive pulmonary embolism [PE] with hemodynamic instability, acute ischemic stroke, and acute myocardial infarction). Furthermore, regular (temporary) interruption of the CRI was expected to allow the body to regenerate and maintain its plasma fibrinogen levels above the threshold of 4.4 μM (150 mg/dL), below which the risk of bleeding is known to significantly increase [2].

After evaluating the benefit–risk ratio of each dosing regimen in Part 1, the DMC recommended to proceed with the high dose in Part 2.

## Measures implemented to prevent clot formation

For study Parts 1 and 2, the infusion scheme (12-hour infusion, followed by a ~12-hour break) was repeated daily from Day 1 up to maximum Day 5, preferably starting at the same time each day. An interruption period of up to 72 hours was allowed in the event of a decrease in fibrinogen of <150 mg/dL, or a bleeding event. If these interruptions extended beyond the protocol-recommended 12-hour alteplase infusion-free interval, the time windows for subsequent infusions moved accordingly, and the last infusion was given later than Day 5 in the study.

To prevent the formation of new clots, patients in the alteplase group received one of two anticoagulant regimens, depending on the investigator’s choice. The first regimen, suitable for patients with an estimated glomerular filtration rate ≥30 mL/min/1.73m^2^, was administration of low-dose low-molecular-weight heparin daily in prophylactic doses, subcutaneously, at the end of the alteplase infusion. The type and dose of low-molecular-weight heparin was at the investigator’s discretion. The second regimen, suitable for all patients irrespective of their estimated glomerular filtration rate, was administration of unfractionated heparin (e.g. 10 IU/kg body weight/h) concomitantly with the long-term alteplase intravenous infusion (to be started immediately after the initial 2-hour alteplase infusion), with a target activated partial thromboplastin time of 1.0- to 1.5-fold the upper limit of normal (ULN) according to the local laboratory. Alternatively, unfractionated heparin could be administered subcutaneously (up to 5000 IU, twice daily). From Day 5 onwards, i.e. post-treatment, either low-molecular-weight heparin or unfractionated heparin was administered to patients in both the alteplase and standard of care (SOC) arms.

## Treatments included in SOC

SOC included any supportive measures applied in hospital, for example the use of ventilation support, hemodynamic support, as well as medical therapies commonly used in patients suffering from severe hypoxemic respiratory failure. Guidance published by the World Health Organization (WHO) at the time recommended systemic corticosteroids for patients hospitalized with COVID-19 and requiring supplemental oxygen [3, 4].

## Inclusion/exclusion criteria

The inclusion criteria included: age ≥18 years (or above legal age); severe hypoxemic respiratory failure with arterial oxygen partial pressure (PaO_2_)/fractional inspired oxygen (FiO_2_) ratio (or estimation of PaO_2_/FiO_2_ ratio from pulse oximetry [oxygen saturation/FiO_2_ ratio]) >100 and ≤300; SARS-CoV-2 positive (laboratory-confirmed reverse transcription polymerase chain reaction test); fibrinogen level ≥lower limit of normal; D-dimer ≥3-fold ULN (Part 1) (modified to ≥1-fold ULN in Part 2) according to local laboratory; and written or verbal informed consent in accordance with Good Clinical Practice, and subject to Ethics Committee approval and local regulations prior to inclusion in the trial.

Exclusion criteria included confirmed massive PE with hemodynamic instability, or any PE (suspected or confirmed, e.g. through CT angiography) that was expected to require therapeutic doses of anticoagulants during the treatment period. Other exclusion criteria included: an indication for therapeutic dosing of anticoagulants; IMV for longer than 48 hours; and a history of chronic pulmonary disease, primary pulmonary arterial hypertension, bleeding disorder or intracranial hemorrhage in the past 6 months.

## Statistical analyses

As this was an open-label study, the detailed statistical analysis plans were finalized prior to patient recruitment in Parts 1 and 2. Sample size calculations were performed using R Version 3.6.1 and nQuery Version 4.0, and statistical analyses were performed using SAS Version 9.4. For description of the data, categorical variables are expressed as count per proportion of group (n, %), continuous variables as mean (± standard deviation) or median (Q1, Q3), and time to event endpoints are presented graphically using Kaplan–Meier plots.

For the purpose of statistical analyses, the two parts of the trial were treated independently (Part 1: descriptive; Part 2: confirmatory). A prespecified interim analysis of data from Part 1 was scheduled to occur after randomization of around 60 patients. Part 2 consisted of two cohorts (patients not on invasive ventilation and IMV), which were analyzed separately. As findings from Part 1 indicated a trend towards a greater effect of alteplase treatment in patients who were not on invasive ventilation versus those receiving IMV (data not shown), Part 2 implemented a prespecified hierarchical testing procedure for the null hypothesis in the cohort of patients who were not on invasive ventilation. Testing of the primary efficacy endpoint was followed by the key secondary endpoints of treatment failure and all-cause mortality.

The focus was on the hypothetical estimand, and deaths were censored at Day 28 as this represented irreversible deterioration (WHO Clinical Progression Scale score 10). In the event of death, the length of a patient’s hospital stay was automatically recorded as 28 days, and the number of oxygen-free days was zero. Patients with early clinical improvement and hospital discharge without observed Day-28 data had their last observed values carried forward.

Part 1 of the study was not powered, and patients were randomized 1:1:1, with a view to explore the data in a descriptive manner. The analysis of Part 2 included a 2-sided, type I error level of alpha=5%, with a power of at least 85% to detect clinically relevant differences between the groups. The randomization ratio of 2:1 thus corresponded to around 140 patients not on invasive ventilation in the alteplase group and around 70 patients not on invasive ventilation in the SOC group. An additional 50 patients in the IMV patient cohort were planned, but not part of the confirmatory testing. Patients were randomized 2:1 (Figure 1) with the aim of treating more patients with the active treatment during the pandemic.

In both parts of the study, the full analysis set was evaluated, consisting of all randomized patients with at ≥1 baseline and ≥1 post-baseline assessment relating to the primary endpoint. Randomization in both parts was stratified by ventilation status (not on invasive ventilation, IMV). Based on analysis of Part 1 data, D-dimer status (<5, ≥5 ULN) was an additional stratification factor in Part 2.

For the primary endpoint in both study parts, a Cox proportional hazards model was used to estimate the hazard ratio (HR) for alteplase versus SOC. From the model, 95% confidence intervals (CIs) and corresponding Wald p-values for the HRs were produced. In Part 1, the model included fixed effects for treatment (alteplase, SOC) ventilation status (not on invasive ventilation/IMV) and age (continuous). In Part 2, the model included the same fixed effects, plus D-dimer status. For the cohort of patients not on invasive ventilation, the number of days of non-invasive ventilation support (see methods section of main manuscript for definition) was also used, and for the IMV patient cohort, the baseline WHO status. From the Kaplan–Meier estimates stratified by treatment at various time points (Days 6, 8, 12, 16, 20, 24 and 28), the risk difference with 95% CI was estimated using the difference of the individual probabilities and the 95% CIs using the variances determined from the Greenwood method. Pooled analyses were additionally stratified by study part (1 or 2). Adjustments were made for treatment, baseline D-dimer status, age, baseline ventilation status and Part 1 or 2 of the study (analysis of PaO_2_/FiO_2_ ratio was also adjusted for baseline PaO_2_/FiO_2_ ratio). For the secondary endpoints of all-cause mortality and treatment failure, the risk difference was estimated between the two treatment groups using logistic regression and the average marginal effect method, adjusting for the same set of factors as the primary endpoint. For the secondary endpoint of change in PaO_2_/FiO_2_ ratio at baseline to Day 6, the mean difference was estimated between the two groups using analysis of covariance adjusted for the same set of factors as the primary endpoint and the baseline PaO_2_/FiO_2_ ratio value. The unadjusted risk differences for the secondary safety endpoint are determined from the Chan and Zhang method.

Pooled analyses, although prespecified, were exploratory. For all individual and pooled analyses in this publication, Part 2 definitions were used if different to Part 1. In particular, this relates to the PaO_2_/FiO_2_ ratio and the detailed definition of ‘treatment-emergent’. Parts 1 and 2 of the study analyzed daily average and worst daily PaO_2_/FiO_2_ ratio values, respectively. However, for the pooled analysis, worst daily PaO_2_/FiO_2_ ratio value was used for both parts. For Part 1, treatment-emergent adverse events (AEs) were any AEs reported from start of alteplase treatment (or from randomization for SOC group) up to 12 days thereafter. In Part 2, any AEs that started before first drug administration and deteriorated under treatment were also considered as ‘treatment-emergent’. Also, AEs in the SOC group were not reported as no investigational medicinal product (alteplase) was administered. For the pooled analysis, TEAEs include any AEs reported from the first administration of alteplase until 288 hours after administration of the first dose or 168 hours after administration of the last dose; for patients on SOC, TEAEs include any AEs reported from the time of randomization until 288 hours.

## Statistical analyses: Adjustments

Following the early discontinuation of the study, three types of analysis (in addition to the protocol-defined analyses) were performed with respect to the pooling of data (Table S5). All analyses contain pooled data from Parts 1 and 2, but supplementary analysis 2 was restricted to the patient cohort not on invasive ventilation and on high-dose alteplase, following the positive findings from Part 1.

The default set of covariates for adjustment in this pooled set of analyses were:

- Ventilation status (patients who were not on invasive ventilation and those who were on IMV) – except for supplementary analysis 2 (Table S10), since this analysis comprised patients who were not on invasive ventilation, with a WHO Clinical Progression Scale score of 6 only
- D-dimer status (<5, ≥5-fold ULN)
- Age (continuous)
- Study part (Part 1, Part 2)

Ventilation status was a randomization stratification factor in both parts, whilst D-dimer status was a randomization stratification factor in Part 2 only, following observations from Part 1. Both are included in the default list of covariates for adjustment in this pooled set of analyses.

Age was prespecified in the default list of covariates for adjustment and again in the pooled set of analyses.

Study part was integral to the study design in this operationally seamless study, and was therefore also included in the default list for the pooled set of analyses.

The number of days of non-invasive ventilation support was prespecified as an adjustment factor for supplementary analysis 2, but since this was not routinely recorded in Part 1, it was not possible to include it in supplementary analysis 2.

No analysis was performed limited to the IMV patient cohort, so there was no adjustment for the baseline WHO Clinical Progression Scale score on the lowest level of granularity (7, 8, 9), which would not have been robust due to the poor/unequal distribution across the treatments.

Lastly, endpoints for which a baseline measurement was recorded included the baseline value in the analysis, for example, analysis of the change in PaO_2_/FiO_2_ ratio from baseline to Day 6 contained the baseline value of the PaO_2_/FiO_2_ ratio.

Sensitivity analyses of the primary endpoint included adjustment for sex (results not shown) and time since diagnosis (with and without study part). Neither had an impact on the results (Table S13).

## Sensitivity analyses of key secondary endpoints

The key secondary endpoints were analyzed using frequency tables, risk differences, 95% CIs and p-values. The delta method and average marginal effect method were used.

## Definition of major bleeding

Major bleeds were defined according to the International Society on Thrombosis and Hemostasis definition [5] and included 1) symptomatic bleeding in a critical area or organ, such as intracranial, intraspinal, intraocular, retroperitoneal, intra-articular or pericardial, or intramuscular with compartment syndrome, and/or 2) bleeding associated with a reduction in hemoglobin of at least 2 g/dL (1.24 mmol/L) or leading to transfusion of two or more units of blood or packed cells. Bleeding should be overt, and the hemoglobin drop should be considered to be due to and temporally related to the bleeding event.

## Figure S1. Study design


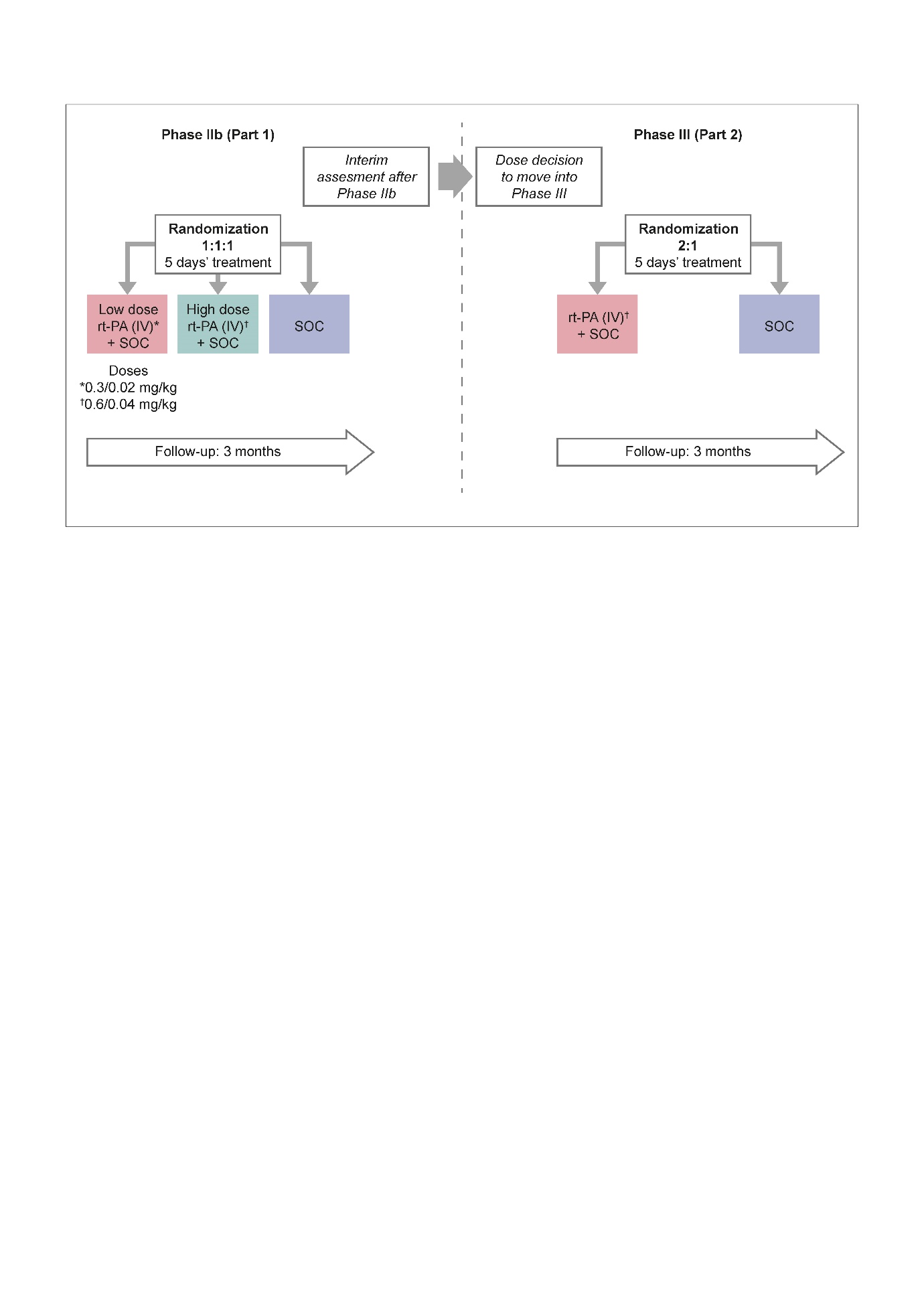


SOC includes supportive measures applied in hospital, such as the use of non-invasive or invasive ventilation, hemodynamic support (if needed), oxygen masks, sedation, as well as medical therapies commonly used in patients suffering from severe hypoxemic respiratory failure or its complications. SOC follows the best possible treatment regimen established locally in line with treatment guidelines for severe hypoxemic respiratory failure.

Follow-up: All-cause mortality at Day 90 (3 months) was predefined as a further endpoint.

IV, intravenous; rt-PA, recombinant tissue-plasminogen activator; SOC, standard of care.

## Table S3. FiO_2_ ranges for common oxygen delivery devices

| **Device** | **Flow (L/min)** | **FiO_2_** |
| --- | --- | --- |
| Nasal cannula | 1–6 | 0.24–0.40 |
| Simple mask | 5–10 | 0.35–0.50 |
| Non-rebreather mask | 10–15 | 0.60–0.80 |
| OxyMask | 1.5–15 | 0.25–0.80 |

## Table S4. Values to derive PaO_2_/FiO_2_ ratio from SpO_2_

| **Measured SpO_2_ (%)** | **Imputed PaO_2_ (mmHg)** |
| --- | --- |
| 100* | 167* |
| 99* | 134* |
| 98* | 104* |
| 97* | 91* |
| 96 | 82 |
| 95 | 76 |
| 94 | 71 |
| 93 | 67 |
| 92 | 64 |
| 91 | 61 |
| 90 | 59 |
| 89 | 57 |
| 88 | 55 |
| 87 | 53 |
| 86 | 51 |
| 85 | 50 |
| 84 | 49 |
| 83 | 47 |
| 82 | 46 |
| 81 | 45 |
| 80 | 44 |
| 79 | 43 |
| 78 | 42 |
| 77 | 42 |
| 76 | 41 |
| 75 | 40 |
| 74 | 39 |
| 73 | 39 |
| 72 | 38 |
| 71 | 37 |
| 70 | 37 |

*Generally considered unreliable on the basis of the sigmoidal shape of the hemoglobin-oxygen dissociation curve.

## Table S5. Summary of cohorts used in statistical analyses

| **Analysis** | **Study part** | **Treatments included** | **Baseline ventilation status** | **Baseline ventilation status** | **Age (continuous)** | **Baseline D-dimer status (<5, ≥5-fold ULN)** | **Study part (1, 2)** | **Number of days on NIV support** | **Baseline  WHO score** | **Baseline P/F ratio** |
| --- | --- | --- | --- | --- | --- | --- | --- | --- | --- | --- |
|  |  |  |  | **Fixed effects for main analysis of primary endpoint and secondary endpoint ACM + treatment as main effect** | | | | | | **Secondary endpoint: P/F ratio** |
| Main  (n=104) | Pooled 1 & 2 | Alteplase pooled (low and high) vs SOC | Pooled NIV and IMV cohorts | Yes | Yes | Yes | Yes | Not applicable for IMV patients | Baseline ventilation status used instead and for IMV, data too sparse | Yes |
| Supplementary analysis 1 (n=104) | Pooled 1 & 2 | Alteplase low vs alteplase high vs SOC | Pooled NIV and IMV cohorts | Yes | Yes | Yes | Yes | Not applicable for IMV patients | Baseline ventilation status used instead and for IMV, data too sparse | Yes |
| Supplementary analysis 2 (n=57) | Pooled 1 & 2 | Alteplase high dose vs SOC | NIV cohort only | No | Yes | Yes | Yes | Not routinely recorded in Part 1, so not possible to use it for the pooled analysis | Not relevant since only one value=6 | Yes |

IMV, invasive mechanical ventilation; NIV, non-invasive ventilation; P/F, arterial oxygen partial pressure/fractional inspired oxygen; SOC, standard of care; ULN, upper limit of normal; WHO, World Health Organization.

#

# Results

# Part 1 results

## Primary endpoint

The primary endpoint (clinical improvement of ≥2 points on the 11-point WHO Clinical Progression Scale or hospital discharge by Day 28) was prespecified as exploratory and was not designed to show statistical significance. Patients in the high-dose alteplase group had a greater likelihood of clinical improvement, with Kaplan–Meier analysis (unadjusted for covariates) showing separation between the high-dose alteplase group and both the low-dose alteplase and SOC-only groups from approximately Day 11. Subgroup analysis of the primary endpoint indicated a clinically important and statistically significant benefit for patients not on invasive ventilation at baseline for high-dose alteplase compared with SOC only.

## Secondary endpoints

Both alteplase groups had lower all-cause mortality than the SOC group at Day 28. Both groups also had a lower risk of treatment failure (all-cause mortality or mechanical ventilation at Day 28) than the SOC-only group. Patients in the high-dose alteplase group had more ventilator-free days from the start of treatment to Day 28 than the low-dose alteplase and SOC groups. In addition, the high-dose alteplase group had a greater improvement in median daily average PaO_2_/FiO_2_ ratio from baseline to Day 6 compared with the low-dose alteplase and SOC-only groups.

In patients not on invasive ventilation, all secondary endpoints (all-cause mortality at Day 28, treatment failure at Day 28, number of ventilator-free days from start of treatment to Day 28 and daily average PaO_2_/FiO_2_ ratio change from baseline to Day 6) showed better clinical outcomes for patients in the high-dose alteplase group compared with the low-dose alteplase and SOC groups (data not shown).

## AEs

The proportions of patients with any AEs, severe AEs and serious AEs were similar between the three treatment groups. The proportions of patients with AEs considered to be drug-related were higher in the high-dose alteplase group than in the low-dose alteplase group.

More patients in the high-dose alteplase group reported AEs that led to discontinuation of study treatment, and major bleeding events up to Day 6 were also more frequent in the high-dose alteplase group than in the low-dose alteplase group.

In patients not on invasive ventilation at baseline, major bleeding events up to Day 6 were more frequent in the high-dose alteplase group than in the low-dose alteplase group (data not shown).

# Pooled results (Parts 1&2)

## All-cause mortality at Day 90

All-cause mortality up to Day 90 was 17/69 (25%) in the alteplase group versus 14/35 (40%) in the SOC group (unadjusted risk difference: –15% [95% CI: –35% to 4%]; p=0.116), with similar findings after adjustment for baseline D-dimer/ventilation status, age, treatment, and study part (risk difference: –14% [95% CI: –33% to 4%]; p=0.134) (main paper, Table 2). There was no difference in risk of all-cause mortality at Day 90 between the high-dose alteplase group (11/49 [22%]; adjusted risk difference vs. SOC: –15% [95% CI: –35% to 5%]; p=0.141) and low-dose group (6/20 [30%]; adjusted risk difference vs. SOC: –12% [95% CI: –37% to 12%], p=0.322; Table S7). In patients not on invasive ventilation, all-cause mortality at Day 90 was 5/33 (15%); adjusted risk difference vs. SOC: –27% [95% CI: –50% to –3%], p=0.027; Table S10).

## Figure S2. Time to clinical improvement or hospital discharge up to Day 28 (Parts 1 and 2, alteplase pooled vs. standard of care)


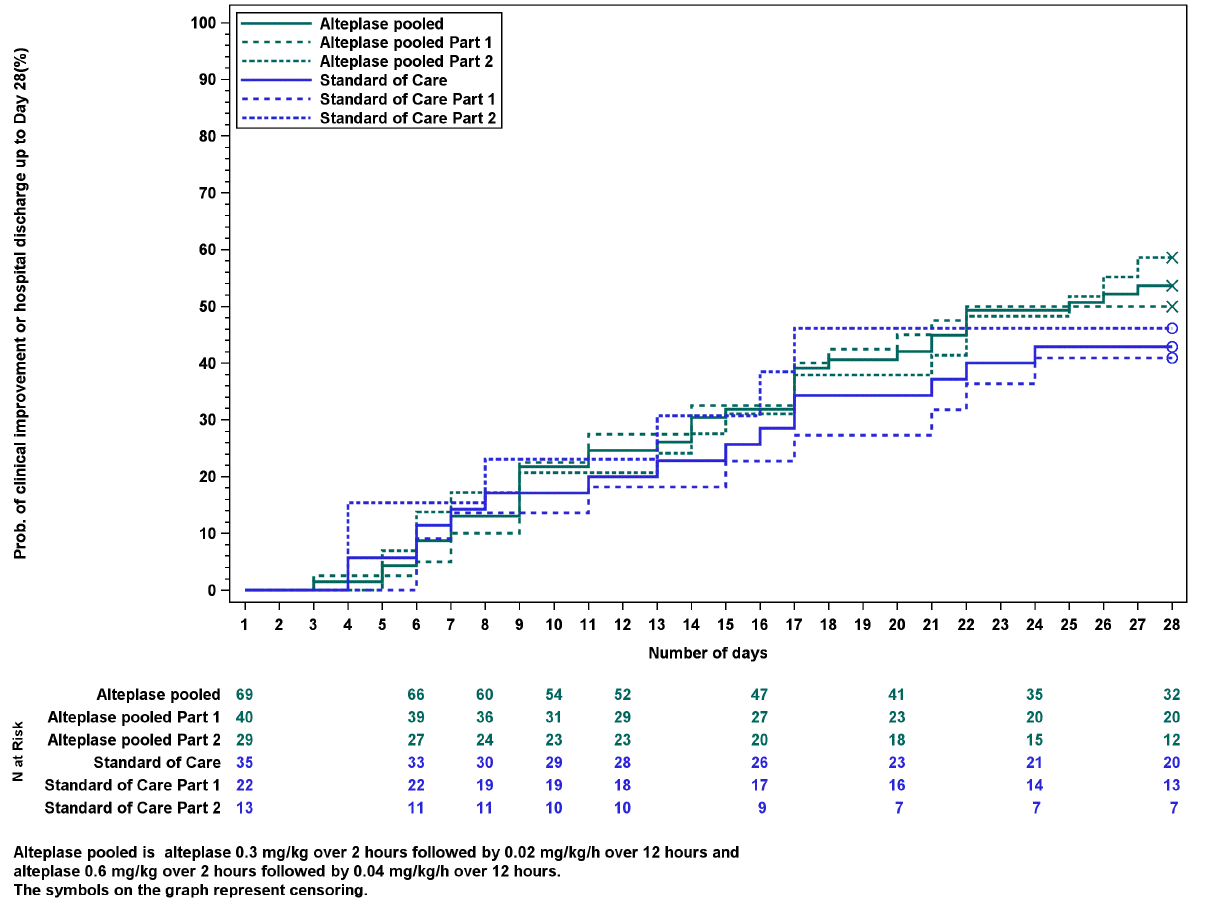


Alteplase pooled includes alteplase Low-dose: 0.3 mg/kg over 2 hours, followed by daily long-term (12-hour) infusion of 0.02 mg/kg/h over 5 days (added to SOC) and High-dose (HD) alteplase: 0.6 mg/kg over 2 hours, followed by daily long-term (12-hour) infusion of 0.04 mg/kg/h over 5 days (added to SOC).

The symbols on the graphs represent censoring.

N, number of patients

# Supplementary analysis 1 (Subgroup 1): Patients stratified according to alteplase dose (Parts 1 and 2 pooled)

## Figure S3. Patient population: all patients (Parts 1 and 2), stratified by dose

**
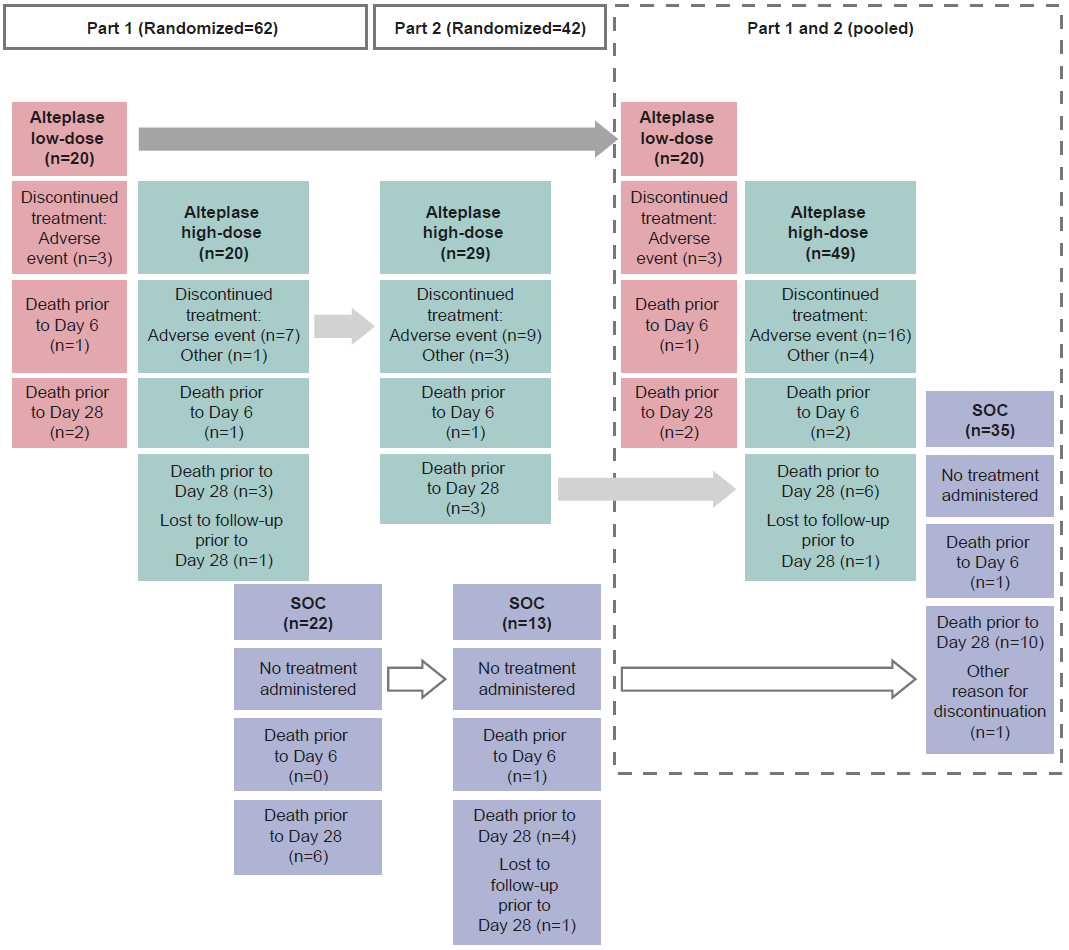
**

Alteplase low dose: 0.3 mg/kg over 2 hours, followed by daily long-term (12-hour) infusion of 0.02 mg/kg/h over 5 days (added to SOC). Alteplase high dose: 0.6 mg/kg over 2 hours, followed by daily long-term (12-hour) infusion of 0.04 mg/kg/h over 5 days (added to SOC). SOC, standard of care.

## Figure S4. Time to clinical improvement up to Day 28: all patients (Parts 1 and 2 vs SOC), stratified by dose


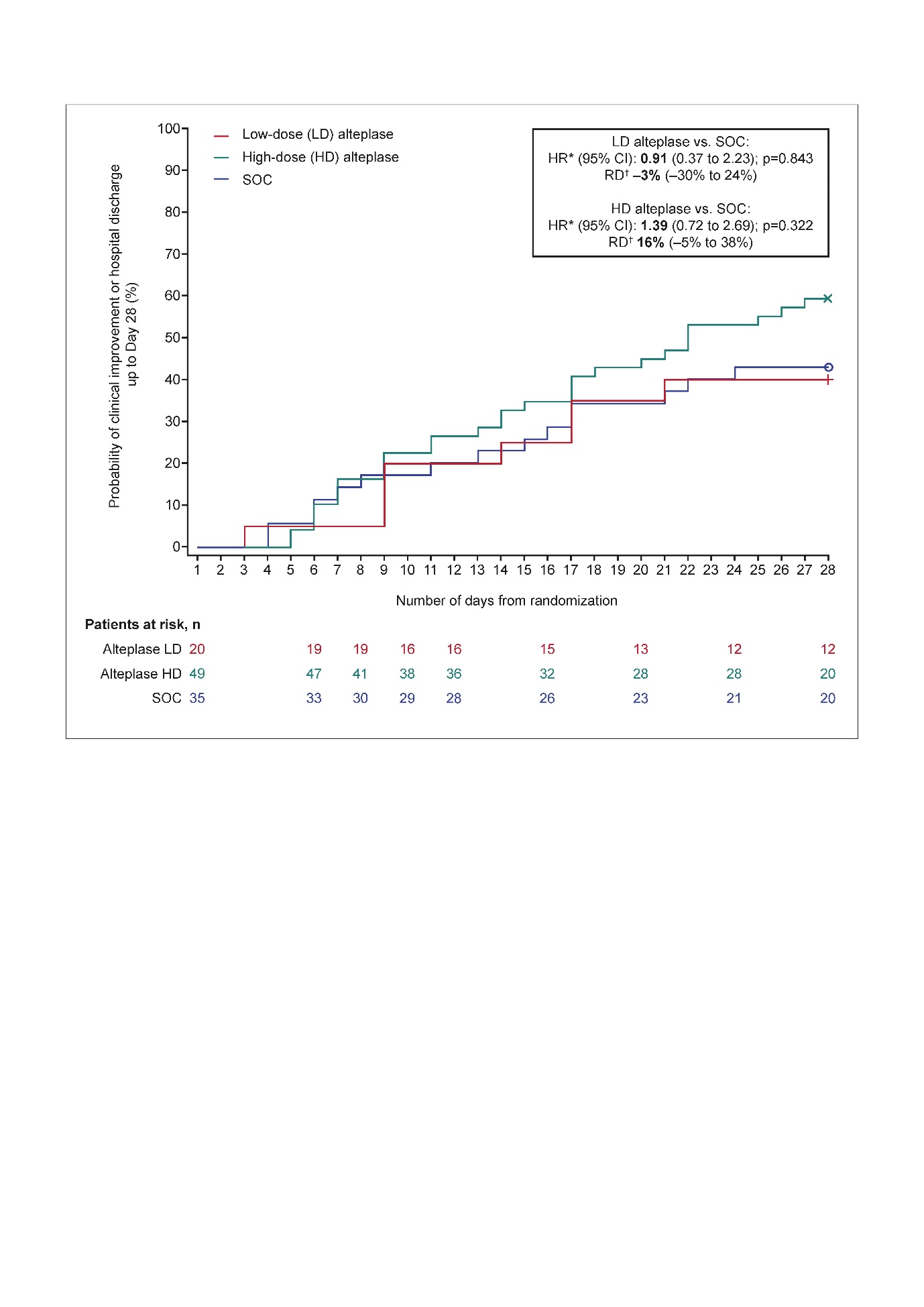


Low-dose (LD) alteplase: 0.3 mg/kg over 2 hours, followed by daily long-term (12-hour) infusion of 0.02 mg/kg/h over 5 days (added to SOC). High-dose (HD) alteplase: 0.6 mg/kg over 2 hours, followed by daily long-term (12-hour) infusion of 0.04 mg/kg/h over 5 days (added to SOC).

*HRs adjusted for treatment, baseline D-dimer status, age, baseline ventilation, and Part 1 or 2 of the study.

†RD corresponds to Day 28

The symbols on the graphs represent censoring.

CI, confidence interval; HD, high dose; HR, hazard ratio; LD, low dose; RD, risk difference; SOC, standard of care.

## Table S6. Baseline characteristics: all patients (Parts 1 and 2), stratified by dose

|  | **Alteplase low dose** | **Alteplase high dose** | **SOC** |
| --- | --- | --- | --- |
| **Patients, n (%)** | 20 (100.0) | 49 (100.0) | 35 (100.0) |
| **Age (years) mean (SD)** | 61.0 (12.0) | 61.7 (10.6) | 61.4 (12.2) |
| **Male, n (%)** | 10 (50.0) | 42 (85.7) | 20 (57.1) |
| **Race*, n (%)**  White  Other  Missing | 13 (65.0)  0  7 (35.0) | 30 (61.2)  2 (4.1)  17 (34.7) | 18 (51.4)  3 (8.6)  14 (40.0) |
| **BMI (kg/m^2^) mean (SD)** | 31.5 (5.1) | 30.1 (5.3) | 29.8 (3.8) |
| **Time since diagnosis (days) mean (SD)** | 11.6 (6.0) | 8.3 (6.9) | 8.7 (5.2) |
| **Smoking status, n (%)**  Never  Former  Current  Missing | 16 (80.0)  3 (15.0)  0  1 (5.0) | 33 (67.3)  14 (28.6)  2 (4.1)  0 | 24 (68.6)  8 (22.9)  0  3 (8.6) |
| **SOFA total score, mean (SD)** | 4.6 (2.3) | 4.8 (2.3) | 4.6 (2.2) |
| **Baseline PaO_2_/FiO_2_ ratio (worst daily value), median (Q1, Q3)** | 109.4 (96.3, 155.0) | 122.7 (104.3, 160.0) | 125.8 (105.3, 166.5) |
| **WHO scale, n (%)**  Score of 6  Score of 7  Score of 8  Score of 9 | 16 (80.0)  1 (5.0)  2 (10.0)  1 (5.0) | 33 (67.3)  2 (4.1)  8 (16.3)  6 (12.2) | 24 (68.6)  6 (17.1)  1 (2.9)  4 (11.4) |
| **Supportive care type, n (%)**  Oxygen by mask or nasal prongs  Oxygen by high flow mask or nasal cannula  Non-invasive ventilation  Invasive mechanical ventilation  Missing | 0  7 (35.0)  9 (45.0)  4 (20.0)  0 | 1 (2.0)  17 (34.7)  14 (28.6)  16 (32.7)  1 (2.0) | 1 (2.9)  7 (20.0)  14 (40.0)  9 (25.7)  4 (11.4) |
| **Concomitant therapy**  Dexamethasone  Tocilizumab (IL-6 inhibitor) | 14 (70.0)  0 | 38 (77.6)  6 (12.2) | 28 (80.0)  4 (11.4) |
| **D-dimer status, N (%)**  ≥ULN to <3-fold ULN  3 to <5-fold ULN  ≥5-fold ULN  Missing | 0  9 (45.0)  11 (55.0)  0 | 2 (4.1)  20 (40.8)  26 (53.1)  1 (2.0) | 1 (2.9)  11 (31.4)  23 (65.7)  0 |

Alteplase low dose: 0.3 mg/kg over 2 hours, followed by daily long-term (12-hour) infusion of 0.02 mg/kg/h over 5 days (added to SOC). Alteplase high dose: 0.6 mg/kg over 2 hours, followed by daily long-term (12-hour) infusion of 0.04 mg/kg/h over 5 days (added to SOC).

*Data on race were not recorded in France (the largest recruiter of patients in this trial).

BMI, body mass index; FiO_2_, fractional of inspired oxygen; IL, interleukin; PaO_2_, arterial oxygen partial pressure; SD, standard deviation; SOC, standard of care; SOFA, Sequential (sepsis-related) Organ Failure Assessment; ULN, upper limit of normal; WHO, World Health Organization.

## Table S7. Primary, secondary and safety endpoints: all patients (Parts 1 and 2), stratified by dose

|  | **Alteplase low dose**  **N=20** | ***p-value*** | **Alteplase high dose N=49** | ***p-value*** | **SOC alone**  **N=35** |
| --- | --- | --- | --- | --- | --- |
| **Primary endpoint** |  |  |  |  |  |
| **Time to clinical improvement* up to Day 28**  Median days to clinical improvement (95% CI)  Patients with event within 28-day timeframe, n (%)  HR vs. SOC (95% CI)  Unadjusted  Adjusted†  Risk difference vs. SOC (95% CI) | NR (14 to NR)  8 (40)  0.91 (0.39 to 2.14)  0.91 (0.37 to 2.23)  –3% (–30 to 24) | 0.828  0.843 | 22 (17 to NR)  29 (59)  1.49 (0.80 to 2.77)  1.39 (0.72 to 2.69)  16% (–5 to 38) | 0.214  0.322 | NR (17 to NR)  15 (43)  −  − |
| **Key secondary endpoints** |  |  |  |  |  |
| **Treatment failure (all-cause mortality or mechanical ventilation) up to Day 28**  Patients with event, n (%)  Risk difference vs. SOC (95% CI)  Unadjusted  Adjusted† | 8 (40)  –9% (–36 to 19)  –8% (–35 to 19) | 0.536  0.570 | 19 (39)  –10% (–31 to 12)  –7% (–29 to 14) | 0.371  0.494 | 17 (49)  −  − |
| **All-cause mortality up to Day 28**  Patients with event, n (%)  Risk difference vs. SOC (95% CI)  Unadjusted  Adjusted† | 2 (10)  –19% (–39 to 1)  –18% (–37 to 1) | 0.068  0.069 | 6 (12)  –16% (–34 to 1)  –14% (–32 to 3) | 0.068  0.101 | 10 (29)  −  − |
| **Other secondary endpoints** |  |  |  |  |  |
| **PaO_2_/FiO_2_ ratio (worst daily value) change from baseline up to Day 6**  Mean ± SD, mmHg  Mean difference vs. SOC (95% CI)  Unadjusted  Adjusted^‡^ | 3.1 ± 75  9 (–34 to 51)  0 (–41 to 41) | 0.693  0.990 | 41.5 ± 86  49 (16 to 83)  43 (11 to 74) | 0.005  0.010 | –11.7 ± 59  −  − |
| **Length of hospital stay up to Day 28^¶^**  Mean ± SD, days  Mean difference vs. SOC (95% CI)  Unadjusted  Adjusted† | 24.1 ± 6.2  –0.3 (–4 to 4)  0.1 (–4 to 4) | 0.876  0.958 | 22.6 ± 7.7  –2 (–5 to 1)  –2 (–5 to 1) | 0.230  0.304 | 24.4 ± 5.9  −  − |
| **Number of oxygen-free days up to Day 28^¶^**  Mean ± SD, days  Mean difference vs. SOC (95% CI)  Unadjusted  Adjusted† | 4.8 ± 7.2  0.3 (–4 to 5)  –0.1 (–5 to 4) | 0.878  0.965 | 7.4 ± 8.7  3 (–1 to 6)  3 (–1 to 6) | 0.097  0.156 | 4.5 ± 7.1  −  − |
| **Further endpoint** | | | | | |
| **All-cause mortality up to Day 90**  Patients with event, n (%)  Risk difference vs. SOC (95% CI)  Unadjusted  Adjusted† | 6 (30)  –10% (–36 to 16)  –12% (–37 to 12) | 0.448  0.322 | 11 (22)  –18% (–38 to 2)  –15% (–35 to 5) | 0.085  0.141 | 14 (40)  −  − |
| **Safety endpoint** |  |  |  |  |  |
| **Major bleeding event up to Day 6**  Patients with event, n (%)  Risk difference vs. SOC (95% CI) | 1 (5)  5% (–6 to 25) |  | 8 (16)  16% (4 to 30) | NA | 0 (0)  − |

Alteplase low dose: 0.3 mg/kg over 2 hours, followed by daily long-term (12-hour) infusion of 0.02 mg/kg/h over 5 days (added to SOC). Alteplase high dose: 0.6 mg/kg over 2 hours, followed by daily long-term (12-hour) infusion of 0.04 mg/kg/h over 5 days (added to SOC).

*Improvement of ≥2 points on the 11-point WHO Clinical Progression Scale, or discharge from the hospital, whichever came first.

†Adjusted for treatment, baseline D-dimer status, age, baseline ventilation, and Part 1 or 2 of the study.

^‡^Adjusted for treatment, baseline PaO_2_/FiO_2_ ratio, baseline D-dimer status, age, baseline ventilation, and Part 1 or 2 of the study.

^¶^In the event of death, the length of a patient’s hospital stay was automatically recorded as 28 days, and the number of oxygen-free days was zero.

CI, confidence interval; FiO_2_, fractional inspired oxygen; HR, hazard ratio; NA, not available; NR, not reported; PaO_2_, arterial oxygen partial pressure; SD, standard deviation; SOC, standard of care; WHO, World Health Organization.

## Table S8. TEAEs* and bleeding: all patients (Parts 1 and 2), stratified by dose

|  | **Alteplase low dose** | **Alteplase high dose** | **SOC** |
| --- | --- | --- | --- |
| **Number of patients, N (%)** | 20 (100.0) | 49 (100.0) | 35 (100.0) |
| **Any AEs** | 16 (80.0) | 44 (89.8) | 30 (85.7) |
| **Severe AEs** | 7 (35.0) | 17 (34.7) | 16 (45.7) |
| **Investigator-defined, drug-related AEs** | 7 (35.0) | 31 (63.3) | 0 |
| **AEs leading to discontinuation of alteplase** | 3 (15.0) | 15 (30.6) | 0 |
| **Bleeding events** |  |  |  |
| Treatment-emergent bleeding | 6 (30.0) | 29 (59.2) | 4 (11.4) |
| Blood transfusion needed† | 1 (5.0) | 2 (4.1) | 1 (2.9) |
| Major bleeding | 2 (10.0) | 8 (16.3) | 1 (2.9) |
| Fatal**^‡^** | 0 | 0 | 0 |
| Non-major bleeding | 4 (20.0) | 24 (49.0) | 3 (8.6) |
| Fatal**^‡^** | 0 | 0 | 0 |
| **Serious AEs** | 8 (40.0) | 26 (53.1) | 18 (51.4) |
| Resulting in death | 3 (15.0) | 4 (8.2) | 6 (17.1) |
| Life threatening | 2 (10.0) | 7 (14.3) | 8 (22.9) |
| Required or prolonged hospitalization | 3 (15.0) | 9 (18.4) | 9 (25.7) |
| **Other significant AEs according to ICH E3**^§^ | 1 (5.0) | 6 (12.2) | 0 |

Data are n (%). Alteplase low dose: 0.3 mg/kg over 2 hours, followed by daily long-term (12-hour) infusion of 0.02 mg/kg/h over 5 days (added to SOC). Alteplase high dose: 0.6 mg/kg over 2 hours, followed by daily long-term (12-hour) infusion of 0.04 mg/kg/h over 5 days (added to SOC).

*For patients on alteplase, TEAEs include any AEs reported from the first administration of alteplase until 288 hours post administration of the first dose or 168 hours post administration of the last dose. For patients on SOC, TEAEs include any AEs reported from the time of randomization until 288 hours.

**^†^**Whole blood cell or packed red blood cell transfusion.

^‡^Fatal bleeds were defined as a bleeding event that the investigator determined was the primary cause of death or contributed directly to death.

^§^Other significant AEs are non-serious AEs that led to treatment discontinuation.

AE, adverse event; ICH, International Council for Harmonization of Technical Requirements for Pharmaceuticals for Human Use; SOC, standard of care; TEAE, treatment-emergent AE.

# Supplementary analysis 2 (Subgroup 2): Patients not on invasive ventilation (Parts 1 and 2 pooled)

## Figure S5. Patient population: patients not on invasive ventilation (Parts 1 and 2)*


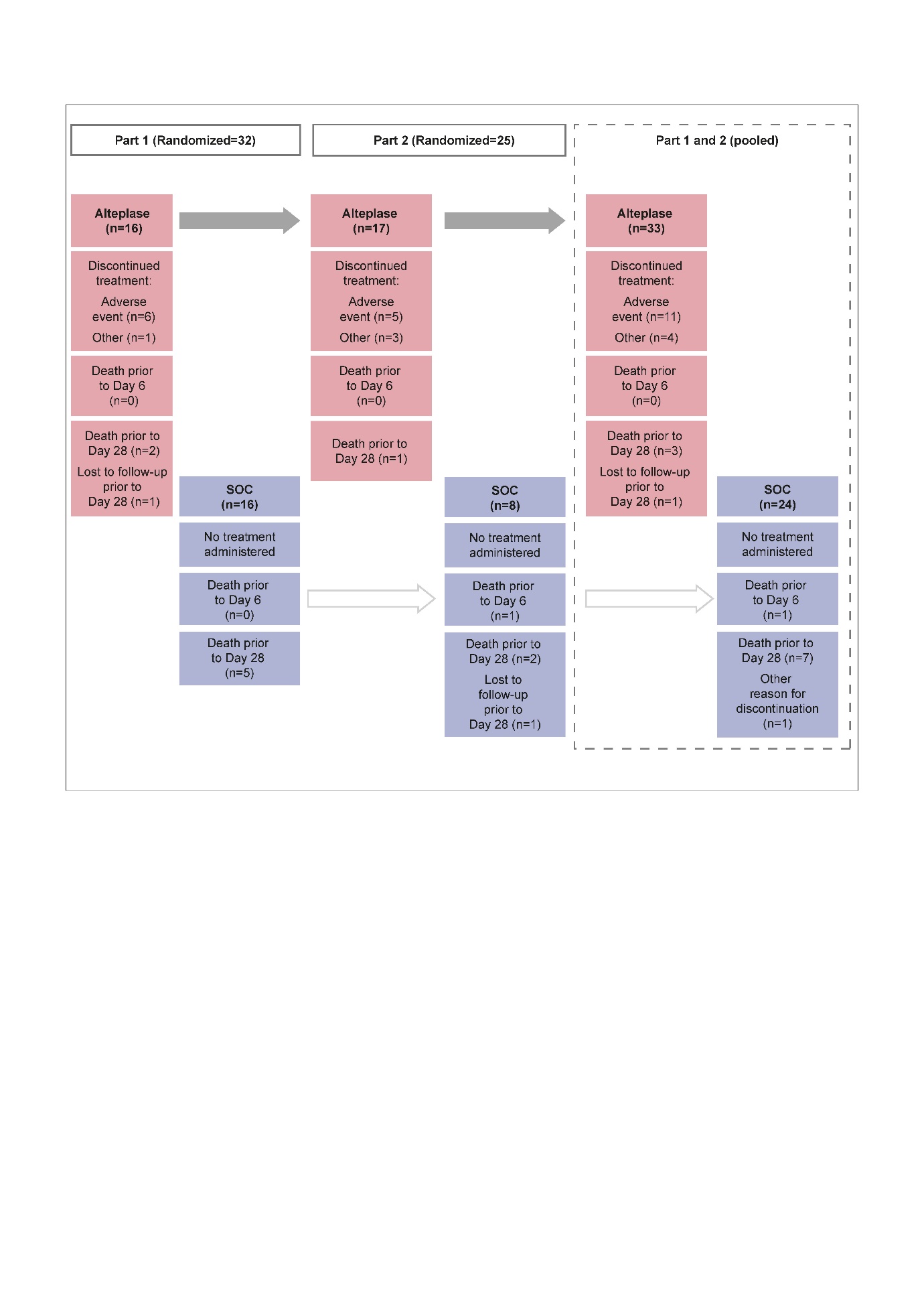


*For Part 1, only patients receiving high-dose alteplase were included in this analysis.

Alteplase high dose: 0.6 mg/kg over 2 hours, followed by daily long-term (12-hour) infusion of 0.04 mg/kg/h over 5 days (added to SOC).

SOC, standard of care.

## Figure S6. Time to clinical improvement or hospital discharge up to Day 28: patients not on invasive ventilation (Parts 1* and 2 vs. SOC, stratified by study part)


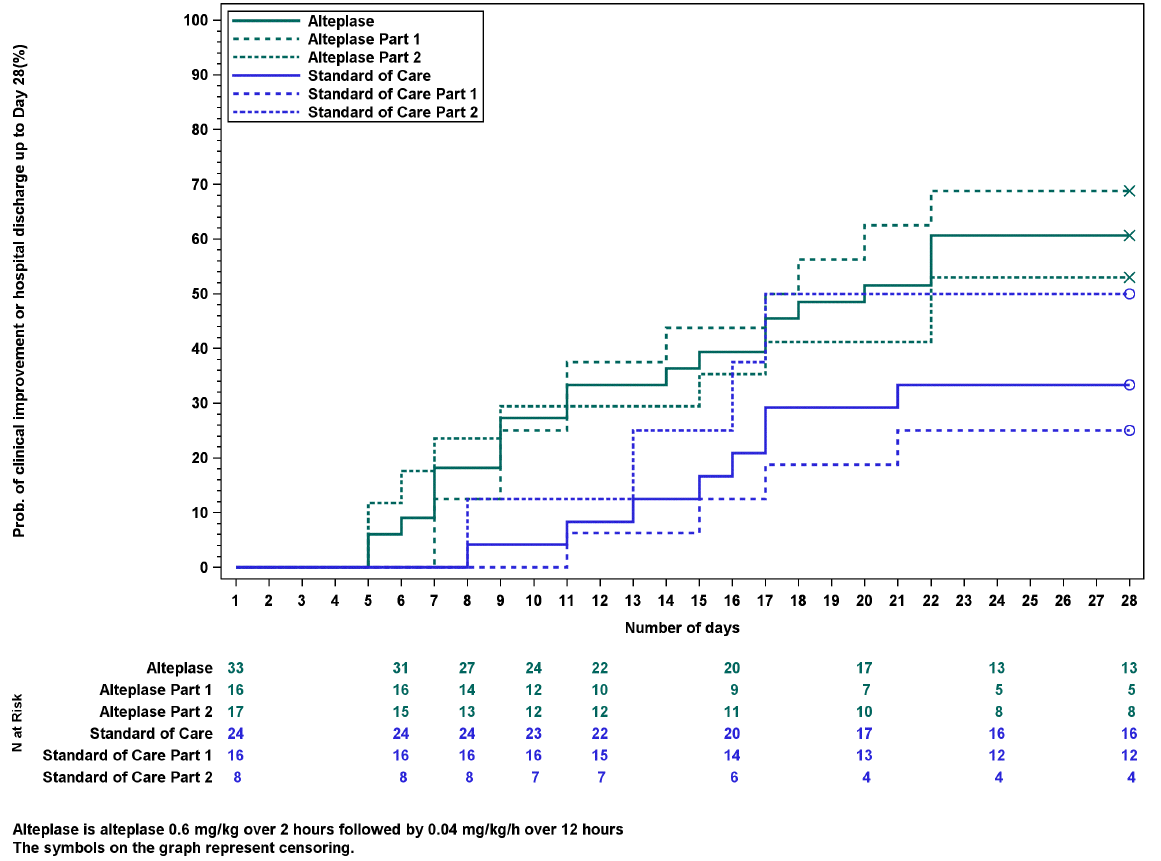


*For Part 1, only patients receiving high-dose alteplase were included in this analysis.

High-dose alteplase: 0.6 mg/kg over 2 hours, followed by daily long-term (12-hour) infusion of 0.04 mg/kg/h over 5 days (added to SOC).

The symbols on the graphs represent censoring.

SOC, standard of care.

## Figure S7. WHO Clinical Progression Scale status at Day 28: patients not on invasive ventilation (Parts 1 and 2 pooled, high-dose alteplase vs. standard of care)


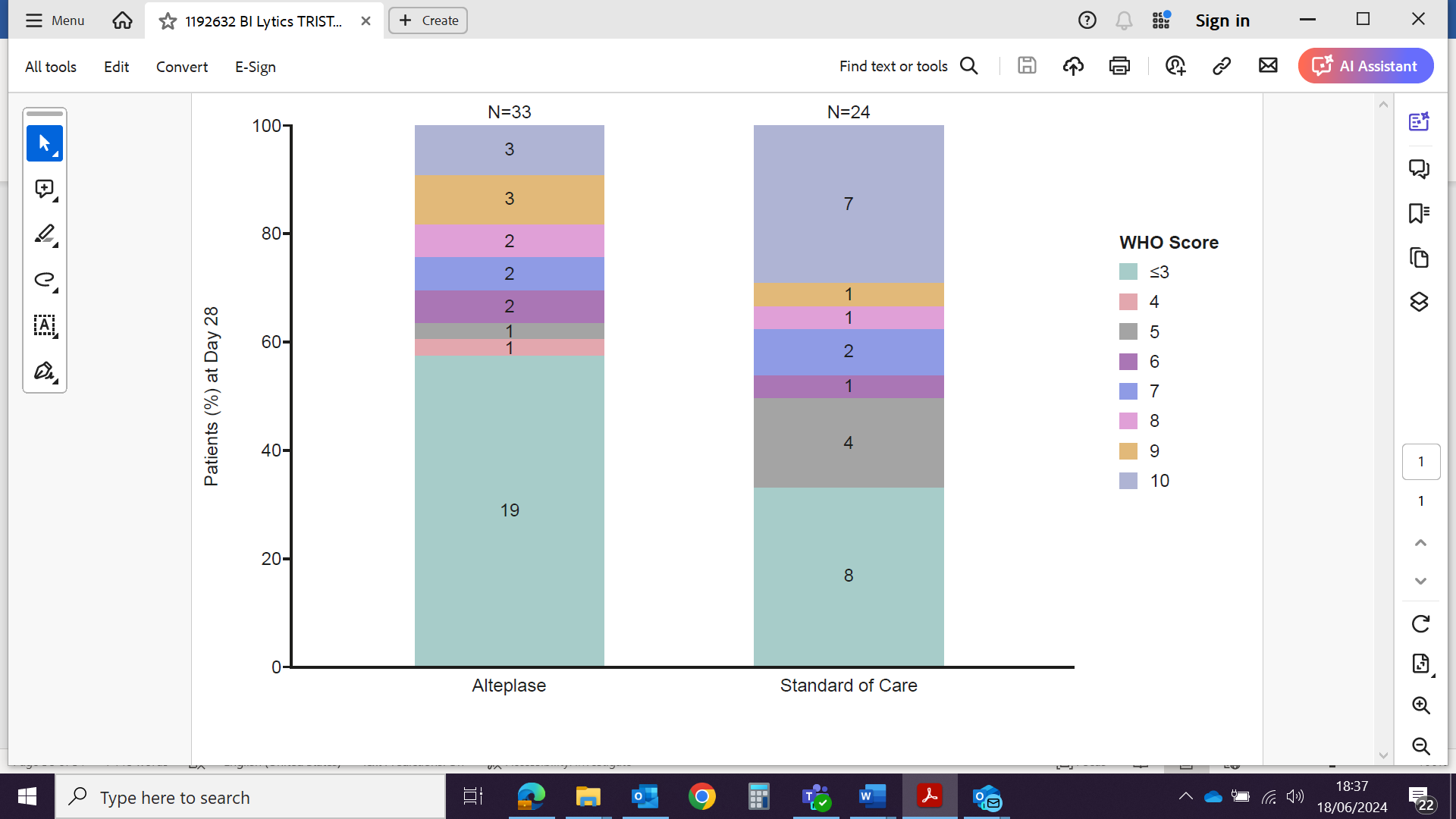


WHO World Health Organization.

## Table S9. Baseline characteristics: patients not on invasive ventilation (Parts 1 and 2)

|  | **Alteplase** | **SOC** | **Total** |
| --- | --- | --- | --- |
| **Number of patients, n (%)** | 33 (100.0) | 24 (100.0) | 57 (100.0) |
| **Age (years) mean (SD)** | 61.9 (10.5) | 59.6 (11.0) | 60.9 (10.7) |
| **Male, n (%)** | 29 (87.9) | 13 (54.2) | 42 (73.7) |
| **Race*, n (%)**  White  Other  Missing | 20 (60.6)  2 (6.1)  11 (33.3) | 15 (62.5)  3 (12.5)  6 (25.0) | 35 (61.4)  5 (8.8)  17 (29.8) |
| **BMI (kg/m^2^) mean (SD)** | 30.1 (5.4) | 30.2 (4.0) | 30.2 (4.8) |
| **Time since diagnosis (days) mean (SD)** | 8.2 (7.4) | 7.8 (5.4) | 8.1 (6.6) |
| **Smoking status, n (%)**  Never  Former  Current  Missing | 24 (72.7)  8 (24.2)  1 (3.0)  0 | 16 (66.7)  5 (20.8)  0  3 (12.5) | 40 (70.2)  13 (22.8)  1 (1.8)  3 (5.3) |
| **SOFA total score, mean (SD)** | 3.6 (1.4) | 3.5 (1.1) | 3.6 (1.3) |
| **Baseline PaO_2_/FiO_2_ ratio (worst daily value), median (Q1, Q3)** | 116.0 (102.5, 136.7) | 123.2 (102.5, 154.8) | 118.3 (102.5, 142.5) |
| **WHO scale, n (%)**  Score of 6  Score of 7  Score of 8  Score of 9 | 33 (100.0)  0  0  0 | 24 (100.0)  0  0  0 | 57 (100.0)  0  0  0 |
| **Supportive care type, n (%)**  Oxygen by mask or nasal prongs  Oxygen by high flow mask or nasal cannula  Non-invasive ventilation  Invasive mechanical ventilation  Missing | 1 (3.0)  17 (51.5)  14 (42.4)  0  1 (3.0) | 1 (4.2)  7 (29.2)  14 (58.3)  0  2 (8.3) | 2 (3.5)  24 (42.1)  28 (49.1)  0  3 (5.3) |
| **Concomitant therapy**  Dexamethasone  Tocilizumab (IL-6 inhibitor) | 25 (75.8)  5 (15.2) | 19 (79.2)  3 (12.5) | 44 (77.2)  8 (14.0) |
| **D-dimer status, n (%)**  ≥ULN to <3-fold ULN  3 to <5-fold ULN  ≥5-fold ULN  Missing | 1 (3.0)  14 (42.4)  17 (51.5)  1 (3.0) | 1 (4.2)  7 (29.2)  16 (66.7)  0 | 2 (3.5)  21 (36.8)  33 (57.9)  1 (1.8) |

Alteplase dose: 0.6 mg/kg over 2 hours, followed by daily long-term (12-hour) infusion of 0.04 mg/kg/h over 5 days (added to SOC).

*Data on race were not recorded in France (the largest recruiter of patients in this trial).

BMI, body mass index; FiO_2_, fractional inspired oxygen; IL, interleukin; PaO_2_, arterial oxygen partial pressure; SD, standard deviation; SOC, standard of care; SOFA, Sequential (sepsis-related) Organ Failure Assessment; ULN, upper limit of normal; WHO, World Health Organization.

##

## Table S10. Primary, secondary and safety endpoints: patients not on invasive ventilation (Parts 1 and 2)

|  | **Alteplase both doses**  **N=33** | **SOC alone**  **N=24** | ***p-value*** |
| --- | --- | --- | --- |
| **Primary endpoint** | | | |
| **Time to clinical improvement* up to Day 28**  Median days to clinical improvement (95% CI)  Patients with event, n (%)  HR vs. SOC (95% CI)  Unadjusted  Adjusted†  Risk difference vs. SOC (95% CI) | 20 (11 to NR)  20 (61)  2.29 (1.01 to 5.20)  2.18 (0.92 to 5.15)  27% (2 to 52) | NR (17 to NR)  8 (33)  −  −  − | 0.048  0.076 |
| **Key secondary endpoints** | | | |
| **Treatment failure (all-cause mortality or mechanical ventilation) up to Day 28**  Patients with event, n (%)  Risk difference vs. SOC (95% CI)  Unadjusted  Adjusted† | 10 (30)  –16% (–41 to 10)  –14% (–39 to 12) | 11 (46)  −  − | 0.230  0.298 |
| **All-cause mortality up to Day 28**  Patients with event, n (%)  Risk difference vs. SOC (95% CI)  Unadjusted  Adjusted† | 3 (9)  –20% (–41 to 1)  –20% (–41 to 1) | 7 (29)  −  − | 0.057  0.058 |
| **Other secondary endpoints** | | | |
| **PaO_2_/FiO_2_ ratio (worst daily value) change from baseline up to Day 6**  Mean ± SD, mmHg  Mean difference vs. SOC (95% CI)  Unadjusted  Adjusted^‡^ | 65 ± 91  78 (35 to 121)  72 (26 to 117) | –13 ± 60  −  − | 0.001  0.003 |
| **Length of hospital stay up to Day 28^¶^**  Mean ± SD, days  Mean difference vs. SOC (95% CI)  Unadjusted  Adjusted† | 20.6 ± 8.4  –3.8 (–8 to 0.2)  –3.8 (–8 to 0.3) | 24.3 ± 5.8  −  − | 0.065  0.067 |
| **Number of oxygen-free days up to Day 28**^¶^  Mean ± SD, days  Mean difference vs. SOC (95% CI)  Unadjusted  Adjusted† | 9.8 ± 9.3  5.5 (1 to 10)  5.3 (1 to 10) | 4.3 ± 6.7  −  − | 0.016  0.023 |
| **Further endpoint** | | | |
| **All-cause mortality up to Day 90**  Patients with event, n (%)  Risk difference vs. SOC (95% CI)  Unadjusted  Adjusted† | 5 (15)  –27% (–50 to –3)  –27% (–50 to –3) | 10 (42)  −  − | 0.025  0.027 |
| **Safety endpoint** | | | |
| **Major bleeding event up to Day 6**  Patients with event, n (%)  Risk difference vs. SOC (95% CI) | 4 (12)  12% (–3 to 28) | 0 (0)  − | NA |

Alteplase dose: 0.6 mg/kg over 2 hours, followed by daily long-term (12-hour) infusion of 0.04 mg/kg/h over 5 days (added to SOC).

*Improvement of ≥2 points on the 11-point WHO Clinical Progression Scale, or discharge from the hospital, whichever came first.

†Adjusted for treatment, baseline D-dimer status, age, and Part 1 or 2 of the study.

^‡^Adjusted for treatment, baseline PaO_2_/FiO_2_ ratio, baseline D-dimer status, age, and Part 1 or 2 of the study.

^¶^In the event of death, the length of a patient’s hospital stay was automatically recorded as 28 days, and the number of oxygen-free days was zero.

CI, confidence interval; FiO_2_, fractional inspired oxygen; HR, hazard ratio; PaO_2_, arterial oxygen partial pressure; SD, standard deviation; SOC, standard of care; WHO, World Health Organization.

## Table S11. TEAEs* and bleeding: patients not on invasive ventilation (Parts 1 and 2)

|  | **Alteplase** | **SOC** |
| --- | --- | --- |
| **Number of patients** | 33 (100.0) | 24 (100.0) |
| **Any AEs** | 28 (84.8) | 20 (83.3) |
| **Severe AEs** | 10 (30.3) | 12 (50.0) |
| **Investigator-defined, drug-related AEs** | 21 (63.6) | 0 |
| **AEs leading to discontinuation of alteplase** | 11 (33.3) | 0 |
| **Bleeding events** |  |  |
| Treatment-emergent bleeding | 19 (57.6) | 2 (8.3) |
| Blood transfusion needed† | 2 (6.1) | 1 (4.2) |
| Major bleeding | 4 (12.1) | 1 (4.2) |
| Fatal**^‡^** | 0 (0) | 0 (0) |
| Non-major bleeding | 16 (48.5) | 1 (4.2) |
| Fatal**^‡^** | 0 | 0 |
| **Serious AEs** | 14 (42.4) | 13 (54.2) |
| Resulting in death | 2 (6.1) | 3 (12.5) |
| Life threatening | 4 (12.1) | 7 (29.2) |
| Required or prolonged hospitalization | 5 (15.2) | 8 (33.3) |
| **Other significant AEs according to ICH E3**^§^ | 5 (15.2) | 0 |

Data are n (%). Alteplase dose: 0.6 mg/kg over 2 hours, followed by daily long-term (12-hour) infusion of 0.04 mg/kg/h over 5 days (added to SOC).

*For patients on alteplase, TEAEs include any AEs reported from the first administration of alteplase until 288 hours post administration of the first dose or 168 hours post administration of the last dose. For patients on SOC, TEAEs include any AEs reported from the time of randomization until 288 hours.

^†^Two patients received a blood transfusion (whole blood cell or packed red blood cell transfusion). Low-dose alteplase, 2 units on Day 13; high-dose alteplase, 1 unit on Day 3.

^‡^Fatal bleeds were defined as a bleeding event that the investigator determined was the primary cause of death or contributed directly to death.

^§^Other significant AEs are non-serious AEs that led to treatment discontinuation.

AE, adverse event; ICH, International Council for Harmonization of Technical Requirements for Pharmaceuticals for Human Use; SOC, standard of care; TEAE, treatment-emergent AE.

## Table S12. TEAEs* leading to discontinuation of alteplase

|  | **Alteplase both doses**  **(N=69)** | **Alteplase low dose**  **(n=20)** | **Alteplase high dose**  **(n=49)** |
| --- | --- | --- | --- |
| **Patients with AEs leading to discontinuation** | 18 (26.1) | 3 (15.0) | 15 (30.6) |
| **Blood and lymphatic system disorders** | 1 (1.4) | 0 (0) | 1 (2.0) |
| Anaemia | 1 (1.4) | 0 (0) | 1 (2.0) |
| Hypofibrogenemia | 1 (1.4) | 0 (0) | 1 (2.0) |
| **Cardiac disorders** | 1 (1.4) | 1 (5.0) | 0 (0) |
| Cardiac arrest | 1 (1.4) | 1 (5.0) | 0 (0) |
| **Gastrointestinal disorders** | 2 (2.9) | 0 (0) | 2 (4.1) |
| Gastrointestinal hemorrhage | 1 (1.4) | 0 (0) | 1 (2.0) |
| Oral hemorrhage | 2 (2.9) | 0 (0) | 2 (4.1) |
| **General disorders and injection site reactions** | 4 (5.8) | 1 (5.0) | 3 (6.1) |
| Catheter site hemorrhage | 3 (4.3) | 1 (5.0) | 2 (4.1) |
| Vessel puncture site hemorrhage | 1 (1.4) | 0 (0) | 1 (2.0) |
| **Musculoskeletal and connective tissue disorders** | 1 (1.4) | 0 (0) | 1 (2.0) |
| Hematoma muscle | 1 (1.4) | 0 (0) | 1 (2.0) |
| **Renal and urinary disorders** | 3 (4.3) | 0 (0) | 3 (6.1) |
| Hematuria | 2 (2.9) | 0 (0) | 2 (4.1) |
| Urinary bladder hemorrhage | 1 (1.4) | 0 (0) | 1 (2.0) |
| **Respiratory, thoracic and mediastinal disorders** | 10 (14.5) | 1 (5.0) | 9 (18.4) |
| Epistaxis | 7 (10.1) | 1 (5.0) | 6 (12.2) |
| Hemoptysis | 1 (1.4) | 0 (0) | 1 (2.0) |
| Pharyngeal hemorrhage | 2 (2.9) | 0 (0) | 2 (4.1) |
| Pulmonary embolism^†^ | 2 (2.9) | 0 (0) | 2 (4.1) |
| **Vascular disorders** | 4 (5.8) | 0 (0) | 4 (8.2) |
| Hematoma | 3 (4.3) | 0 (0) | 3 (6.1) |
| Hemorrhage | 1 (1.4) | 0 (0) | 1 (2.0) |

Data represent number of patients, n (%).

*For patients on alteplase, TEAEs include any AEs reported from the first administration of alteplase until 288 hours post administration of the first dose or 168 hours post-administration of the last dose.

^†^Diagnosed via CT angiography or other investigative procedure.

AE, adverse event; CT, computed tomography; TEAE, treatment-emergent AE.

## Table S13. Sensitivity analyses of key secondary endpoints: all patients (Parts 1 and 2)

|  | **Alteplase both doses**  **N=69** | ***p-value*** | **SOC alone**  **N=35** |
| --- | --- | --- | --- |
| **Treatment failure (all-cause mortality or mechanical ventilation) up to Day 28**  Patients with event, n (%)  Risk difference vs. SOC (95% CI)  Unadjusted  Adjusted*  Sensitivity analysis 1†  Sensitivity analysis 2^‡^ | 27 (39)  –9% (–30 to 11)  –8% (–27 to 12)  –9% (–28 to 10)  –9% (–28 to 10) | 0.359  0.448  0.365  0.350 | 17 (49)  −  −  −  − |
| **All-cause mortality up to Day 28**  Patients with event, n (%)  Risk difference vs. SOC (95% CI)  Unadjusted  Adjusted*  Sensitivity analysis 1†  Sensitivity analysis 2^‡^ | 8 (12)  –17% (–34 to 0)  –16% (–31 to 1)  –16% (–32 to 1)  –16% (–32 to 0) | 0.047  0.058  0.058  0.056 | 10 (29)  −  −  −  − |

Alteplase includes doses: 0.3 mg/kg over 2 hours, followed by daily long-term (12-hour) infusion of 0.02 mg/kg/h over 5 days (added to SOC) or 0.6 mg/kg over 2 hours, followed by daily long-term (12-hour) infusion of 0.04 mg/kg/h over 5 days (added to SOC).

*Adjusted for treatment, baseline D-dimer status, age, baseline ventilation, and Part 1 or 2 of the study.

†Adjusted for treatment, baseline D-dimer status, age, baseline ventilation, Part 1 or 2 of the study, and time since diagnosis.

‡Adjusted for treatment, baseline D-dimer status, age, baseline ventilation, and time since diagnosis.

CI, confidence interval; SOC, standard of care.

## Table S14. Major bleeding events up to Day 6 in patients receiving alteplase^*^

| **Patient** | **Part 1 or 2 of study** | **Alteplase dose** | **AE preferred term** | **Time since randomization** | **Transfusion (Y/N)** |
| --- | --- | --- | --- | --- | --- |
| 1 | Part 1 | Low | Epistaxis | 4 days | N |
| 2 | Part 1 | High | Hemoptysis | 3 days | N |
| 3 | Part 1 | High | Mouth hemorrhage | 4 days | N |
| 4 | Part 1 | High | Muscle hematoma | 5 days | Y |
| 5 | Part 1 | High | Pharyngeal hemorrhage | 1 day | N |
| 6 | Part 2 | High | Hemoptysis | 1 day | N |
| 7 | Part 2 | High | Hemorrhage | 3 days | Y |
| 8 | Part 2 | High | Hemorrhage (urinary tract and trachea) | 3 days, 5 days | N |
| 9 | Part 2 | High | Mouth hemorrhage | 2 days | N |

*All patients survived.

# Supplemental references

1. Characterisation, W.H.O.W.G.o.t.C. and C.-i. Management of, *A minimal common outcome measure set for COVID-19 clinical research.* Lancet Infect Dis, 2020. **20**(8): p. e192-e197.

2. Skeik, N., et al., *Fibrinogen level as a surrogate for the outcome of thrombolytic therapy using tissue plasminogen activator for acute lower extremity intravascular thrombosis.* Vasc Endovascular Surg, 2013. **47**(7): p. 519-23.

3. Group, W.H.O.R.E.A.f.C.-T.W., et al., *Association Between Administration of Systemic Corticosteroids and Mortality Among Critically Ill Patients With COVID-19: A Meta-analysis.* JAMA, 2020. **324**(13): p. 1330-1341.

4. Agarwal, A., et al., *A living WHO guideline on drugs for covid-19.* BMJ, 2020. **370**: p. m3379.

5. Schulman, S., C. Kearon, and Subcommittee on Control of Anticoagulation of the Scientific Standardization Committee of the International Society on Thrombosis and Haemostasis, *Definition of major bleeding in clinical investigations of antihemostatic medicinal products in non-surgical patients.* J Thromb Haemost, 2005. **3**(4): p. 692-694.
